# Absolute quantification of cellular levels of photosynthesis-related proteins in *Synechocystis* sp. PCC 6803

**DOI:** 10.1007/s11120-022-00990-z

**Published:** 2022-12-21

**Authors:** Philip J. Jackson, Andrew Hitchcock, Amanda A. Brindley, Mark J. Dickman, C. Neil Hunter

**Affiliations:** 1 Plants, Photosynthesis and Soil, School of Biosciences, University of Sheffield, Sheffield, S10 2TN UK; 2 Department of Chemical and Biological Engineering, University of Sheffield, Sheffield, S1 3JD UK

**Keywords:** *Synechocystis*, Cyanobacteria, Mass spectrometry, Photosynthesis, Photosystem, Assembly factor, Chlorophyll, Electron transport

## Abstract

Quantifying cellular components is a basic and important step for understanding how a cell works, how it responds to environmental changes, and for re-engineering cells to produce valuable metabolites and increased biomass. We quantified proteins in the model cyanobacterium *Synechocystis* sp. PCC 6803 given the general importance of cyanobacteria for global photosynthesis, for synthetic biology and biotechnology research, and their ancestral relationship to the chloroplasts of plants. Four mass spectrometry methods were used to quantify cellular components involved in the biosynthesis of chlorophyll, carotenoid and bilin pigments, membrane assembly, the light reactions of photosynthesis, fixation of carbon dioxide and nitrogen, and hydrogen and sulfur metabolism. Components of biosynthetic pathways, such as those for chlorophyll or for photosystem II assembly, range between 1000 and 10,000 copies per cell, but can be tenfold higher for CO_2_ fixation enzymes. The most abundant subunits are those for photosystem I, with around 100,000 copies per cell, approximately 2 to fivefold higher than for photosystem II and ATP synthase, and 5–20 fold more than for the cytochrome *b*_6_*f* complex. Disparities between numbers of pathway enzymes, between components of electron transfer chains, and between subunits within complexes indicate possible control points for biosynthetic processes, bioenergetic reactions and for the assembly of multisubunit complexes.

**Supplementary Information:**

The online version contains supplementary material available at 10.1007/s11120-022-00990-z.

## Introduction

Cyanobacteria evolved approximately 2.4 billion years ago as the only prokaryotes that utilize solar energy to oxidize water. Electron transport coupled to proton translocation then drives the production of the ATP and NADPH needed for CO_2_ fixation and other metabolic processes and reviewed by Lea-Smith et al. (2016). Cyanobacteria have colonized almost every terrestrial and aquatic habitat, with marine species alone responsible for an estimated carbon capture rate of 4 × 10^12^ kg y^−1^ (Rousseaux and Gregg 2014), contributing 3.8% of global net primary production (Field et al. 1998). In view of their ecological significance, the effects of the anthropogenic increase in atmospheric CO_2_ on cyanobacterial populations and their influence on the entire biosphere are important areas of study (Ullah et al. 2018).

Some avenues of research are specific for cyanobacteria, but others have wider relevance to photosynthesis in algae and plants due to the common ancestry of cyanobacteria and chloroplasts (Yoon et al. 2004). Similarities between cyanobacterial and eukaryotic photosystems have led to the adoption of several species of cyanobacteria, often thermophiles, as models for exploring the fundamental mechanisms that underpin oxygenic photosynthesis (Ferreira et al. 2004; Umena et al. 2011; Suga et al. 2015; Gisriel et al. 2019; Çoruh et al. 2021). The early availability of a complete genome sequence for *Synechocystis* sp. PCC 6803 (hereafter *Synechocystis*) was also an invaluable resource for studies of photosynthesis and many other cellular functions. This was the first such information for any phototroph, and only the third genome sequence for any organism (Kaneko et al. 1996). This advance, as well as the amenability of this cyanobacterium to genetic manipulation (Vermaas 1994), hastened the adoption of *Synechocystis* as a model for photosynthesis research. One recent example, which builds upon the evolutionary relationship between chloroplasts and cyanobacteria, is the use of *Synechocystis* as a platform for the rapid development of genetic diversity by adaptive evolution. Laboratory-evolved mutations in cyanobacteria such as *Synechocystis* that confer enhanced efficiency for converting solar energy into biomass may then be transferable to crop plants with the aim of increasing yield (Leister 2012; Dann and Leister 2017; Dann et al. 2021). Model cyanobacterial species have also been valuable for designing solar-powered systems for synthetic biology (reviewed by Sengupta et al. 2018).

In view of its universally recognized importance in photosynthesis research, *Synechocystis* has been a frequent target for mass spectrometry (MS)-based proteomic analysis, as reviewed by Gao et al. (2015) and Battchikova et al. (2018). A principal focus has been on the comparative quantification of protein abundance following adaptation to varying culture conditions (for example see Fulda et al. 2000; Hong et al. 2014; Angeleri et al. 2019) and mutant strains have been used as tools for the dissection of adaptive and regulatory pathways (for example see Tokumaru et al. 2018; Krynická et al. 2019). This approach has also mapped proteins to their subcellular locations to track the development of thylakoid membranes (TMs), the specialized photosynthetic membranes that cyanobacteria, algae and plants all have in common (Kwon et al. 2010; Pisareva et al. 2011). More recently, a proteomic catalog of subcellular localization has been produced, comprising 1712 proteins (Baers et al. 2019).

Photosynthesis research is starting to encompass larger and larger structures, from complexes, to supercomplexes, to membrane organization studied by atomic force microscopy and finally to whole cells (MacGregor-Chatwin et al. 2017; Casella et al. 2017; Zhao et al. 2020). Here, spectacular recent advances in cryo-electron tomography, augmented by super-resolution fluorescence imaging, are starting to reveal the molecular details of cyanobacterial cells, their internal cellular components and their membrane architectures (Rast et al. 2019; Huokko et al. 2021). With this focus on cells it is appropriate to count cellular components in terms of the copy numbers of proteins per cell, which would appear to be a basic requirement for understanding cellular function, and for manipulations of cells and their pathways for synthetic biology purposes. Yet despite the considerable volume of proteomic information now available for *Synechocystis* and other cyanobacteria, to our knowledge there has been no MS-based absolute quantification of proteins in terms of copy number per cell (cpc). So far, cpc determination has been confined to photosystems I and II (PSI and PSII) using absorption spectra associated with their bound chlorophylls (Fujita and Murakami 1987; Hihara et al. 1998; Keren et al. 2004; Fraser et al. 2013; MacGregor-Chatwin et al. 2017). MS-derived absolute quantitative studies of proteins are nevertheless commonplace, having been undertaken in many different organisms and subcellular systems. Some examples, employing both stable isotope labelled (SIL) standards and label-free approaches, are: *Escherichia coli* (Wiśniewskia and Rakus 2014), *Leptospira interrogans* (Malmström et al. 2009), chromatophores in *Rhodobacter sphaeroides* (Cartron et al. 2014), glycolytic pathway enzymes in *Saccharomyces cerevisiae* (Carroll et al. 2011) and xenobiotic metabolizing enzymes in human liver microsomes (Li et al. 2015). Two of the SIL-based methods are confined to the absolute quantification of single or low numbers of proteins and involve calibration with either ^15^N-labelled synthetic peptide fragments (usually tryptic) mapping to the target protein(s) (Kirkpatrick et al. 2005) or full-length SIL proteins produced in *E. coli* grown in ^15^N-containing liquid culture (Brun et al. 2007; Singh et al. 2009). For larger scale absolute quantification the high-financial cost of the former and long lead-time of the latter are potential obstacles that make label-free quantification (LFQ) methods more attractive and therefore more widely used. Since no single LFQ method has emerged as the ‘gold standard’, numerous performance comparisons can be found in the literature (for example see Arike et al. 2013; Fabre et al. 2014; Krey et al. 2014; Al Shweiki et al. 2017; Tang et al. 2019).

**Fig. 1:**
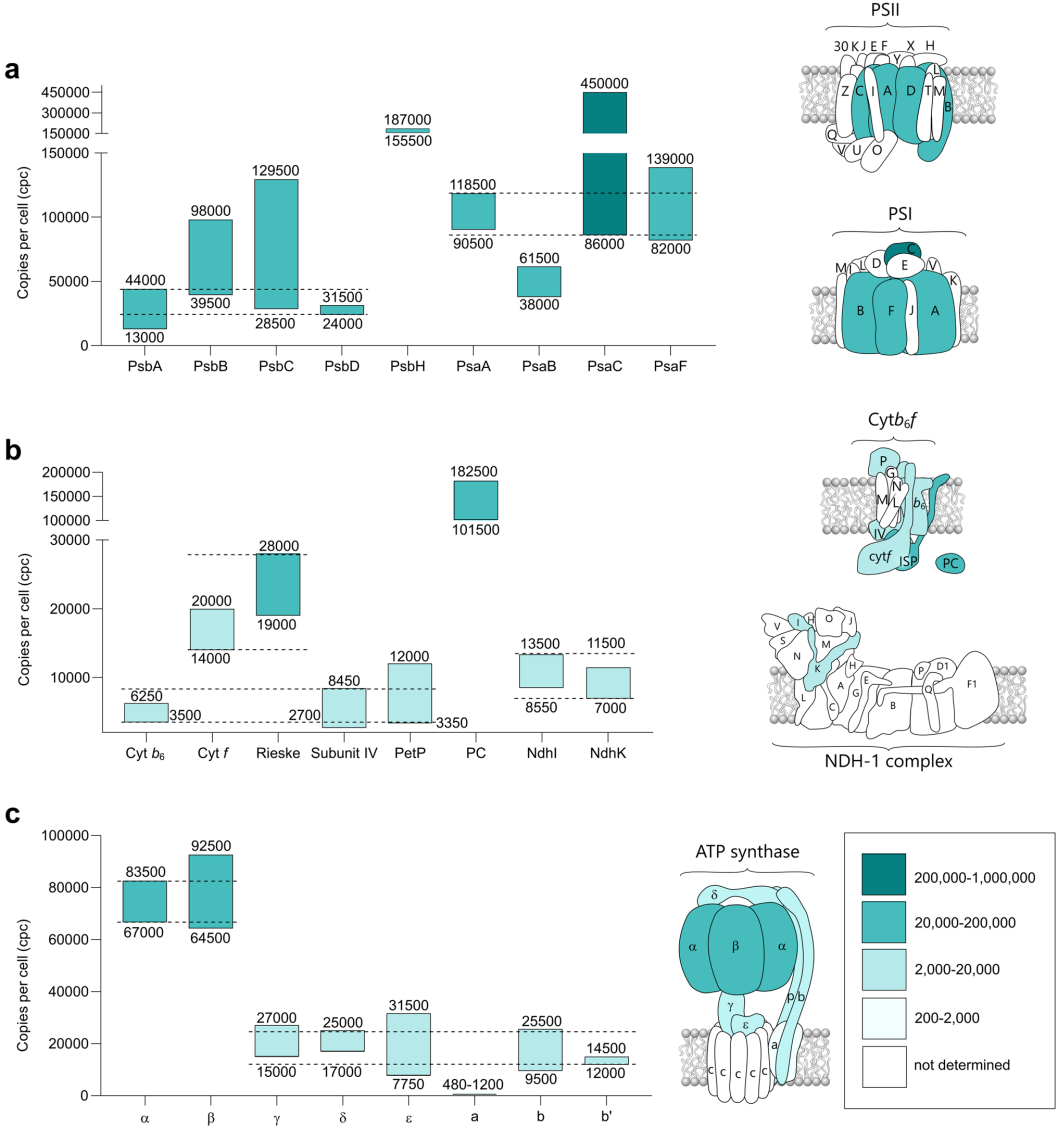
Cellular levels of protein complexes involved in the conversion of solar to chemical energy. Consensus cpc ranges for the subunits are condensed from the different quantification methods, as described in Results and Discussion, with individual data-points shown in boxplots in Supplementary Fig. S2. The minimum and maximum cpc values are rounded to the nearest 10 (< 1000), 50 (1000–10,000) or 500 (> 10,000) and displayed as bars, shaded according to cpc range, for photosystem II (PSII, PsbA-H), photosystem I (PSI, PsaA-F) (**a**), cytochrome *b*_6_*f* (cyt*b*_6_*f*), PetP, phycocyanin (PC), photosynthetic NAD(P)H dehydrogenase-like complex type-1 (NDH-1) (b) and ATP synthase (c). Abundance levels corresponding to the largest extent of overlap between subunits, shown by the horizontal dashed lines, are taken to represent probable ranges for the complexes, explained in Results and Discussion: PSII (24,000–44,000 cpc), PSI (86,000–118,500 cpc), cyt*b*_6_*f* (3350–8450 or 14,000–28,000 cpc), NDH-1 (7000–13,500 cpc), ATP synthase (*α*, *β*: 67,000–83,500 cpc; *γ*, *δ*, *ε*, a, b, b': 12,000–25,000 cpc)

**Fig. 2:**
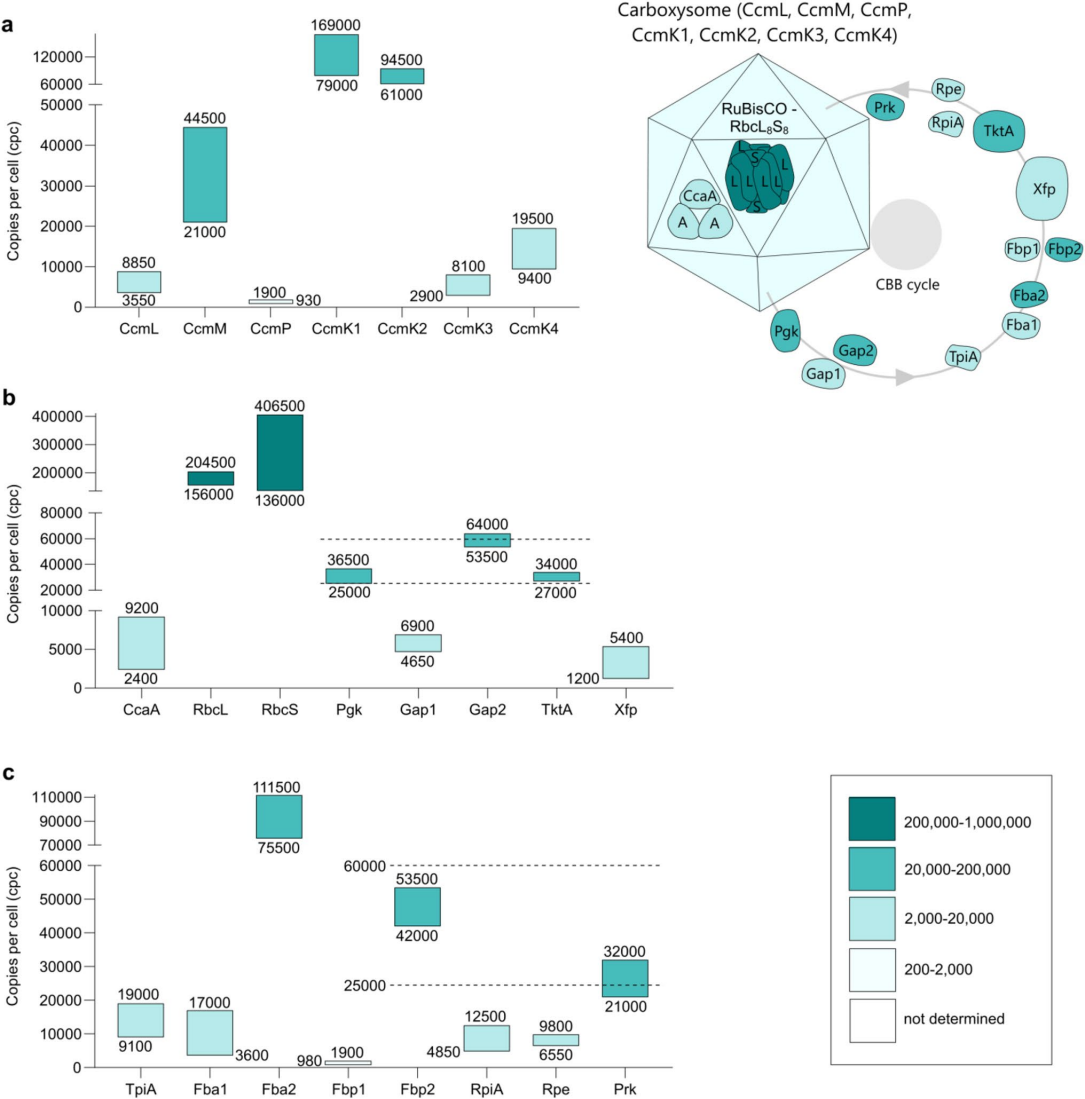
Cellular levels of carboxysomal proteins and enzymes of the Calvin–Benson–Bassham cycle. Consensus cpc ranges are derived from data-points shown in Supplementary Fig. S3 and displayed as in Fig. 1. Structural carboxysomal proteins (**a**), Calvin–Benson–Bassham cycle enzymes: carbonic anhydrase (CcaA), ribulose-1,5-bisphosphate carboxylase-oxygenase (RuBisCO: large, RbcL and small, RbcS subunits), glycerate-3-phosphate kinase (Pgk), glycerate-1,3-phosphate dehydrogenase (Gap1, 2), transketolase (TktA), phosphoketolase (Xfp) (**b**), triose phosphate isomerase (TpiA), fructose-1,6-bisphosphate aldolase (Fba1, 2), fructose-1,6- and sedoheptulose-1,7-bisphosphatase (Fbp1, 2), ribose-5-phosphate isomerase (RpiA), ribulose-3-phosphate epimerase (Rpe), ribulose-5-phosphate kinase (Prk) (**c**). The horizontal dashed lines in (**b**) and (**c**) define a 25,000–60,000 cpc range, explained in Results and Discussion

**Fig. 3:**
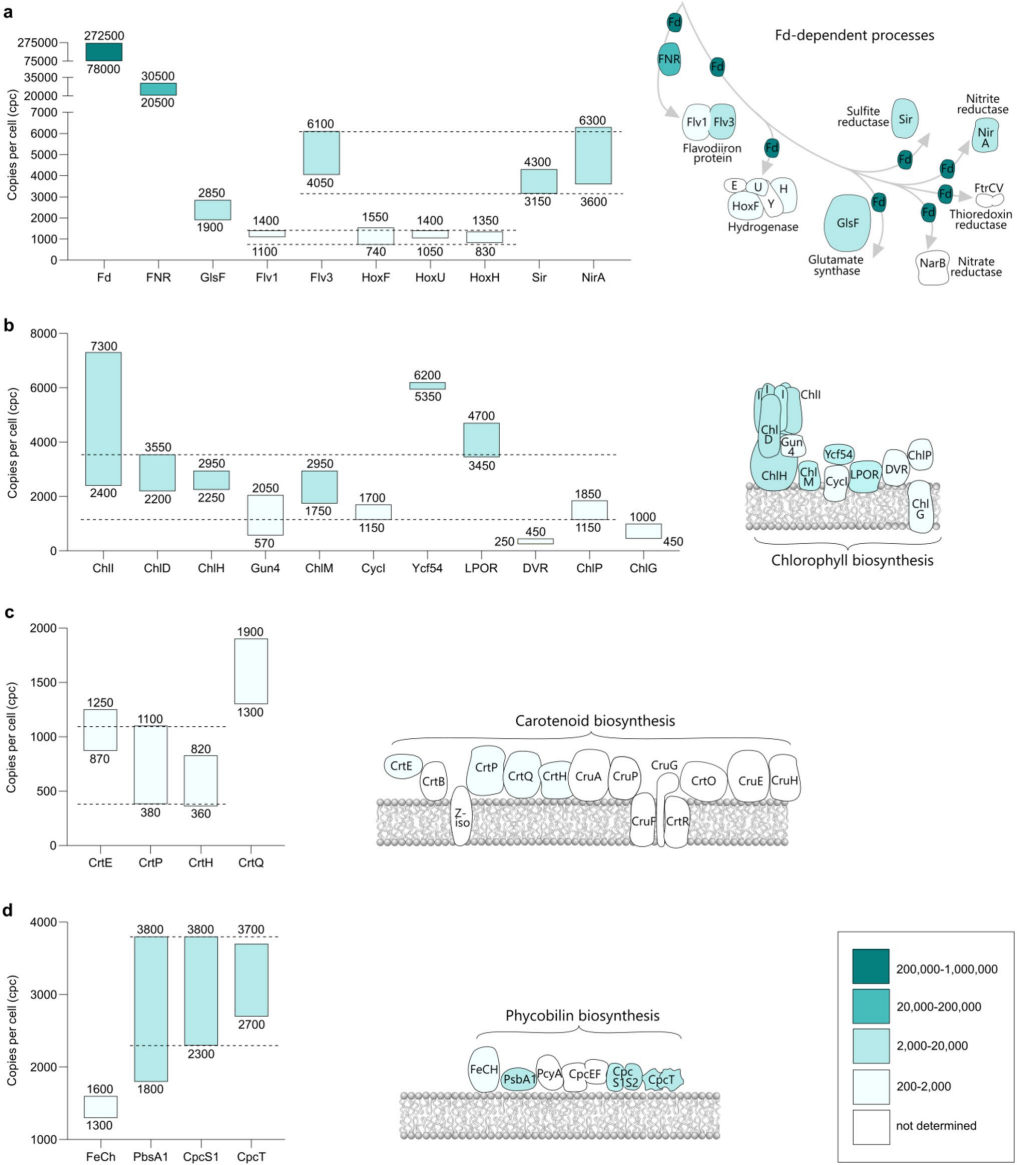
Cellular levels of enzymes and auxiliary proteins involved in biosynthesis. Consensus cpc ranges are derived from data-points shown in Supplementary Fig. S4 and displayed as in Fig. 1. Ferredoxin (Fd) and Fd-dependent enzymes: ferredoxin-NADP ^+^ reductase (FNR), glutamate synthase 2 (GlsF), flavodiiron protein (Flv1/3), bidirectional hydrogenase (HoxF, U, H), sulphite reductase (Sir), nitrite reductase (NirA) (**a**), enzymes and auxiliary proteins in the Mg-branch of the chlorophyll *a* biosynthesis pathway: Mg-chelatase (ChlI, ChlD, ChlH, Gun4), Mg-protoporphyrin IX methyltransferase (ChlM), O_2_-dependent Mg-protoporphyrin IX methyl ester cyclase (CycI, Ycf54), light-dependent protochlorophyllide oxidoreductase (LPOR), 8-vinyl reductase (DVR), geranylgeranyl reductase (ChlP), chlorophyll *a* synthase (ChlG) (**b**), enzymes in the carotenoid biosynthesis pathway: geranylgeranyl pyrophosphate synthase (CrtE), phytoene desaturase (CrtP), prolycopene isomerase/CRTISO (CrtH), ζ-carotene desaturase (CrtQ) (**c**), enzymes in the phycobilin biosynthesis pathway: ferrochelatase (FeCh), heme oxygenase 1 (PbsA1), chromophore lyase (CpcS1, CpcT) (**d**). The horizontal dashed lines, explained in Results and Discussion, define in: (**a)** 740–1400 and 3150–6100 cpc, (**b)** 1150–3550 cpc, (**c)** 380–1100 cpc and (**d)** 2300–3800 cpc

**Fig. 4:**
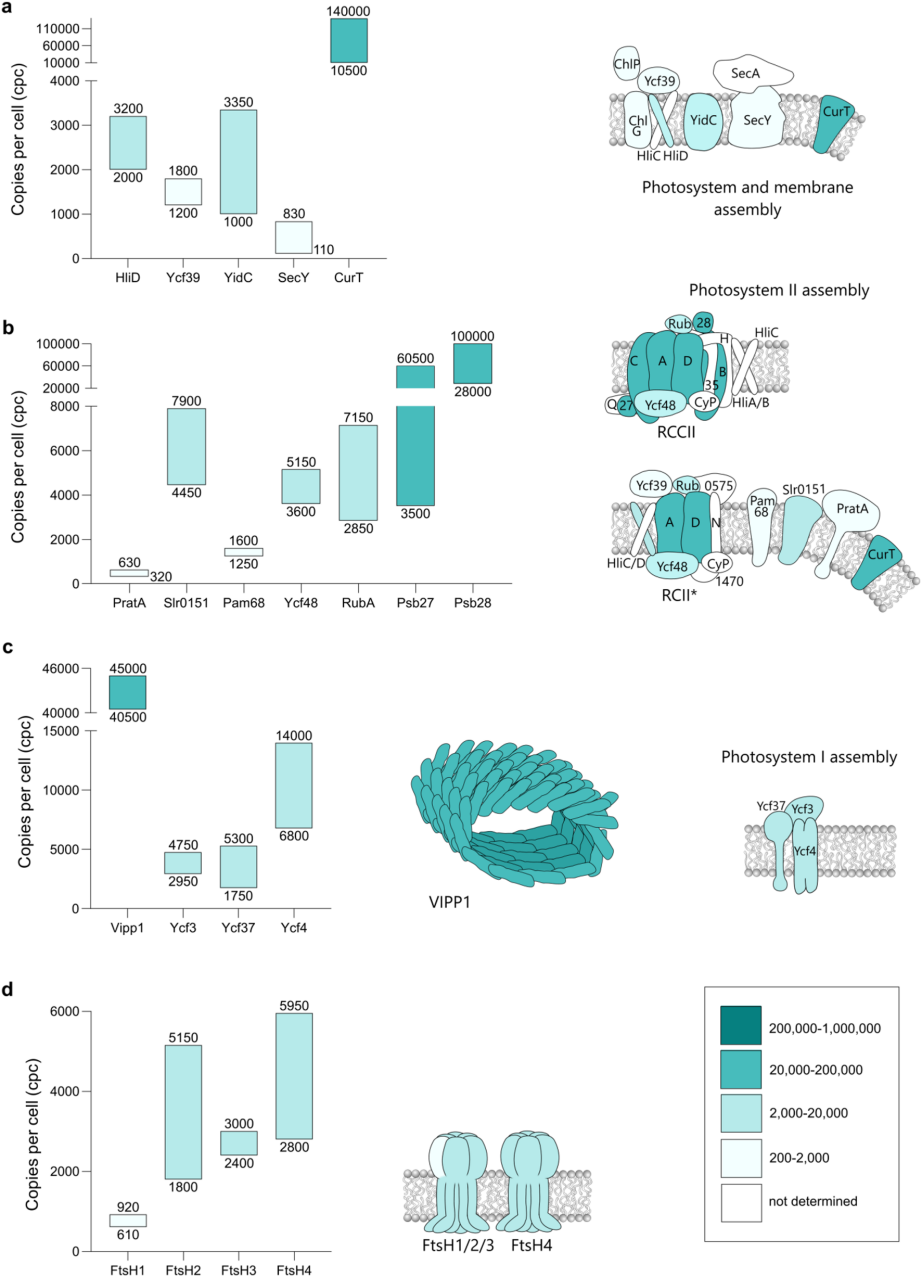
Cellular levels of assembly factors and enzymes involved in thylakoid membrane biogenesis and photosystem assembly/repair. Consensus cpc ranges are derived from data-points shown in Supplementary Fig. S5 and displayed as in Fig. 1. Proteins involved in the coordination of chlorophyll and PSII biosynthesis (**a**), proteins involved in thylakoid membrane biogenesis and PSII assembly (**b**), proteins involved in thylakoid membrane biogenesis and PSI assembly (**c**), and ATP-dependent zinc metalloproteases: membrane protein quality control and PSII repair (**d**)

**Fig. 5:**
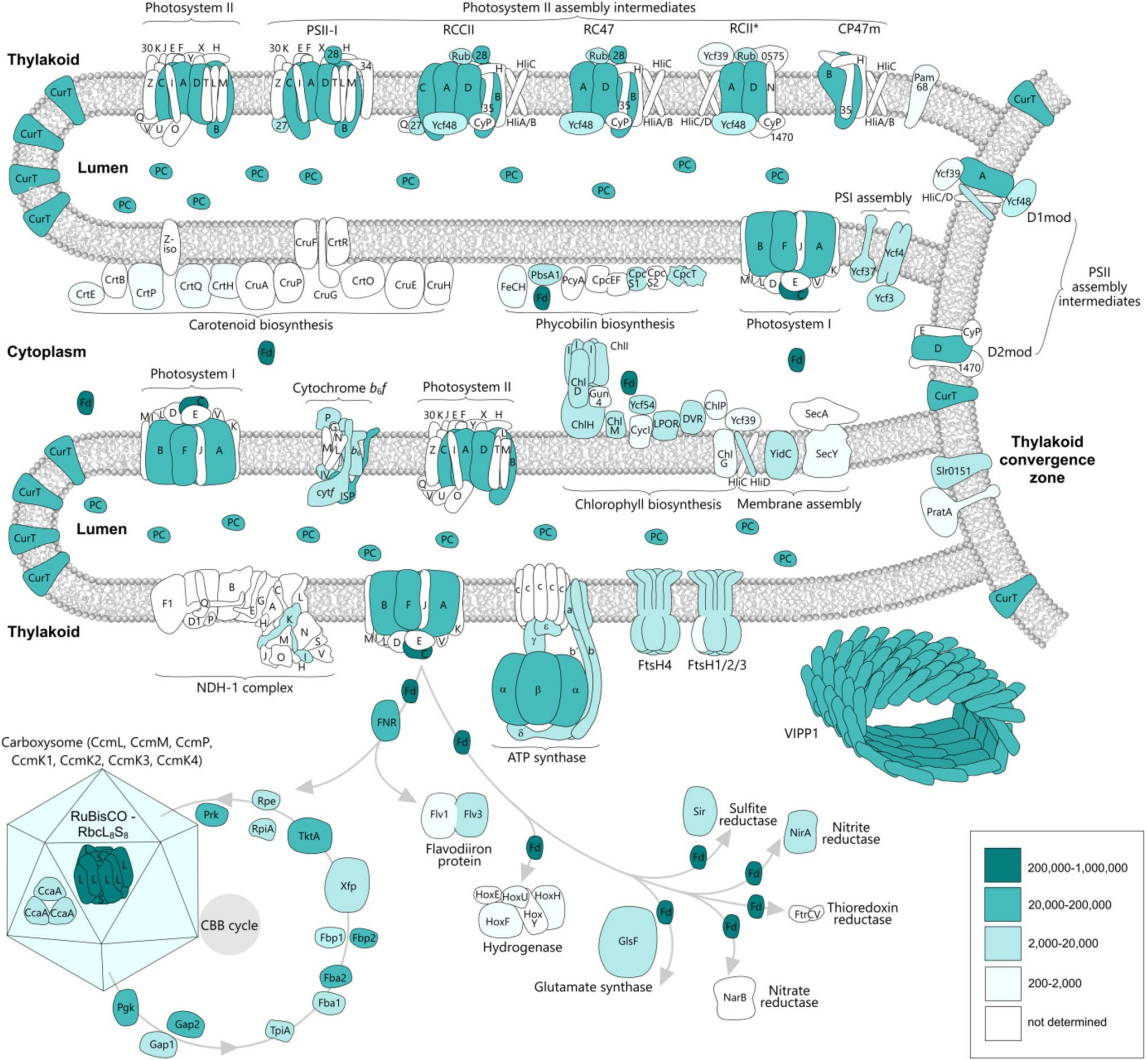
Diagrammatic summary of the proteins quantified in this study, showing the range of biological processes, such as biosynthetic and assembly pathways, covered by the quantitative mass spectrometry analysis. Abbreviations are defined in Figs. 1, 2,3, 4. Proteins and subunits of complexes that have been quantified are colored in blue and shaded according their abundance levels; those not quantified are in white. Complexes such as photosystem I, photosystem II and cytochrome *b*_6_*f* are drawn as monomers for simplicity. Thylakoids are drawn as elongated tubular structures, which converge on a thylakoid convergence zone that appears to connect plasma and thylakoid membranes (Stengel et al. 2012; Heinz et al. 2016). The photosystem II assembly intermediates are based on those in Konert et al. (2022) and Rahimzadeh-Karvansara (2022), with the exception of the PSII-I assembly complex, the structure of which was determined by Zabret et al. (2021)

Here, we employed a third SIL-based quantification method that is more feasible for this larger scale study than the two options described above. It is also well characterized and uses artificial ^15^N-labelled proteins comprising concatenated tryptic peptides mapping to the target proteins (Pratt et al. 2006; Brownridge et al. 2013). To provide further validation and extend the range of target proteins, we additionally employed three LFQ methods: (1) iBAQ (intensity-based absolute quantification) (Schwanhäusser et al. 2011) based on data-dependent acquisition (DDA) and Top3 (Silva et al. 2006), based on both (2) DDA and (3) data-independent acquisition (DIA) (Venable et al. 2004). We demonstrate that our MS-based absolute quantification of PSI and PSII complexes is in close agreement with copy numbers determined spectrophotometrically, both here and in earlier studies (Fujita and Murakami 1987; Hihara et al. 1998; Keren et al. 2004; Fraser et al. 2013; MacGregor-Chatwin et al. 2017). Similarly, we show subunit stoichiometry in the ATP synthase complex aligns with the known structure for the complex in bacteria (Guo et al. 2019). Having validated our approach, we present the first report of MS-based absolute quantification of cytochrome *b*_6_*f* (cyt*b*_6_*f*) subunits, associated mediators of electron transport and downstream electron-accepting metabolic processes including CO_2_ fixation. Also quantified are enzymes and auxiliary proteins in the chlorophyll (Chl), carotenoid and phycobilin biosynthesis pathways, together with assembly factors implicated in TM biogenesis and PSI and PSII assembly. These 97 proteins and their interrelationships are summarized in Figs. 1, 2, 3, 4, 5. Finally, we exploit our quantitative analyses to present the cellular abundances of 1081 proteins in *Synechocystis*.

## Materials and methods

### Cell culture

*Synechocystis* sp. PCC 6803 cells (wild-type substrain GT-P, Tichý et al. 2016) were grown under photoautotrophic conditions in a rotary shaking incubator at 150 rpm under moderate light conditions (30 µmol photons m^−2^ s^−1^) at 30 °C in liquid BG11 medium (Rippka et al. 1979) supplemented with 10 mM TES-KOH (*pH* = 8.2). Three replicate 80 mL cultures were harvested at *OD*_*750*_ = 0.6–0.7 by centrifugation at 5000 x*g* for 20 min at 4 °C. Cells were counted in a hemocytometer after allowing them to settle on the slide for 30 min and stored as pellets at − 80 °C after flash-freezing in liquid nitrogen.

### Preparation of artificial stable isotope-labelled internal standards

Six artificial SIL internal standard proteins comprising concatenated proteotypic tryptic peptide sequences mapping to thirty-seven target proteins were designed as described previously (Pratt et al. 2006; Brownridge et al. 2013) These proteins were expressed in *E. coli* in M9 medium containing (^15^NH_4_)_2_SO_4_ as the sole nitrogen source and purified according to Qian et al. (2013).

### Extraction and digestion of *Synechocystis* proteins for quantification

Cells (5.6 × 10^8^) were solubilized in 100 µL 2% (w/v) sodium dodecylsulfate, 60 mM DL-dithiothreitol by vortexing with 100 µL 0.1 mm silica-zirconia beads according to Krynická et al. (2019). Three samples each corresponding to 1.6 × 10^8^ cells were taken from each of the three replicate lysates and one set of samples was spiked with 15 pmol of each SIL standard protein. Proteins were extracted from all samples then reduced, S-alkylated and digested with pre-mixed trypsin/endoproteinase Lys-C as described by Krynická et al. (2019).

### Analysis by mass spectrometry

Peptides were re-dissolved in 0.1% (v/v) trifluoroacetic acid, 3% (v/v) acetonitrile (LC grade). The samples containing SIL standards were analysed by nano-flow liquid chromatography (3 h gradient) coupled to a mass spectrometer (MS, Q Exactive HF, Thermo Scientific) at 400 ng/injection (three technical repeats of each biological replicate in randomized order). DDA parameters were as specified by Flannery et al. (2021). The remaining two sample sets for LFQ were spiked with a tryptic digest of Universal Protein Standard 2 (UPS2, Sigma-Aldrich), prepared according to the manufacturer’s instructions, so that each 400 ng *Synechocystis* peptide injection was supplemented with 42.4 ng of UPS2 peptides comprising 200 fmol of the highest concentration UPS2 proteins. One of the UPS2 containing sample sets was analysed in triplicate (randomized order) by DDA as above, the other was analysed in triplicate (randomized order) by DIA using the ‘DIA60’ parameters described by Cole et al. (2019).

### Data processing

The SIL raw MS data-files were first converted to Mascot Generic Format (MGF) using the MSConvert tool in ProteoWizard v. 3.0.8934 (Chambers et al. 2012). The MGF files were used as input for database searching by Mascot Daemon v. 2.5.1 running with Mascot Server v. 2.5.1 (Matrix Science) against the *Synechocystis* sp. PCC 6803 Cyanobase proteome database (http://genome.kazusa.or.jp/cyanobase/Synechocystis; latest release 2003, 3672 entries). Search parameters were: enzyme, trypsin; fixed modification, carbamidomethyl (C); variable modification, oxidation (M); maximum missed cleavages, 1; mass tolerances (peptide and fragment), 0.05 Da; quantification, ^15^N metabolic. The search results, in the form of Mascot DAT files were imported into Skyline v. 4.1.0.18169 (MacClean et al. 2010) to generate a spectral library for protein quantification by comparison between the ion intensities of tryptic peptides from *Synechocystis* cells and their ^15^N-labelled counterparts from the internal standards. The peptide peak assignments and ^14^N:^15^N ratio calculations displayed by Skyline were curated manually.

The LFQ-DDA raw MS data-files were processed with MaxQuant v. 1.5.3.30 (Cox and Mann 2008) using the default parameters, with the exceptions that the match between runs and iBAQ (validated by Schwanhäusser et al. 2011) options were enabled. In addition to the *Synechocystis* database mentioned above, the search included a database of the proteins in the UPS2 standard. Identification and quantification results generated by MaxQuant were manipulated in Perseus v. 1.5.3.2 (Tyanova et al. 2016). Quantification of the target proteins was by interpolation of log_10_-transformed iBAQ intensities within regression lines calculated for the UPS2 standard proteins. The LFQ-DDA data set was also used to manually extract quantitative information based on the summed intensities of the three highest intensity tryptic peptides for each target protein, a method referred to as ‘Top3’ (Silva et al. 2006). Calibration was by comparison with ‘Top3’ intensities for the UPS2 standard proteins, as above. The LFQ-DIA raw MS data-files were processed in Skyline with the spectral library created from an imported MaxQuant msms.txt file derived from processing LFQ-DDA MS data, with UPS2 calibration as above. The peptide peak assignments displayed by Skyline were curated manually.

### Chlorophyll *a* determination

*Synechocystis* cells (1.2 × 10^8^, counted as detailed above) were pelleted in a microcentrifuge at 5000 x*g* for 5 min from 1 mL of culture medium (*OD*_*750*_ = 0.65) and extracted with 1 mL of 100% methanol. Chl *a* was quantified by spectrophotometry according to Porra et al. (1989).

## Results and discussion

### Validation of protein identification and quantification

Validation of the cell counting, protein identification and absolute quantification methods are described in Supplementary Information.

### Conversion of solar to chemical energy

#### Photosystem II

PSII is a multi-subunit complex that is integrated into the TM and contains Chl cofactors that enable its photochemical function (Ferreira et al. 2004). The process of oxygenic photosynthesis in *Synechocystis* (reviewed by Lea-Smith et al. 2016) is initiated when solar energy captured by phycobilisomes is transferred to PSII to drive the abstraction of electrons from water, producing O_2_ and releasing protons into the thylakoid lumen.

The four core PSII subunits were quantified: PsbA-D, also referred to as D1, CP47, CP43 and D2 respectively, alongside one of the small subunits, PsbH. Figure 1a shows that the reaction center heterodimer subunits D1 and D2 are closely aligned at 24,000–44,000 cpc, indicated by the two horizontal dashed lines. This range is not only consistent with structural studies establishing the 1:1 stoichiometry of D1 and D2 (Umena et al. 2011; Suga et al. 2015; Gisriel et al. 2022) but also a functional assay of PSII abundance, using flash-induced O_2_ yield in the related *Synechocystis* sp. PCC 6714 (Fujita and Murakami 1987), at 18,000–22,000 cpc. At the lower extent of their abundance range, CP47 and CP43 are within 24,000–44,000 cpc, however at the upper extent these subunits occur at approximately 100,000 cpc, substantially in excess of the one subunit per complex stoichiometry in PSII (Umena et al. 2011; Suga et al. 2015; Gisriel et al. 2022). This observation may be explained by evidence for an excess of the CP47m and CP43m assembly modules over D1- and D2-containing intermediates (Tichý et al. 2016; Bečková et al. 2022), all of which would be included in our whole cell analysis. We therefore suggest that the cellular level of functional PSII complexes in *Synechocystis*, grown under the conditions employed here, is 24,000–44,000 cpc.

Like CP47 and CP43, the 7 kDa subunit PsbH is shown to occur markedly in excess of D1 and D2, at 155,500–187,000 cpc (Fig. 1a). Since PsbH has a stabilizing function, as an obligatory component of both complete, active PSII (Komenda et al. 2002) and several of its assembly intermediates (Komenda 2005), this high level would be expected.

#### Cytochrome *b*_6_*f* complex and plastocyanin

The cyt*b*_6_*f* complex provides the link in the electron transport pathway between PSII and PSI in oxygenic photosynthesis and, like the photosystems, is integral to the TMs (Lea-Smith et al. 2016). In oxygenic phototrophs, this complex comprises four major and four minor subunits (reviewed by Malone et al. 2021). The four major subunits, PetA, PetB, PetC and PetD (cyt *f*, cyt *b*_6_, the Rieske Fe-S protein (ISP) and Subunit IV (subIV) respectively) were quantified at 3500–6250, 14,000–20,000, 19,000–28,000 and 2700–8450 cpc, respectively (Fig. 1b). Despite the known stoichiometry of 1:1:1:1 in the cyanobacterial and plant cyt*b*_6_*f* structures (Malone et al. 2021), these ranges unexpectedly fell into lower (cyt *b*_6_ and subIV) and higher (cyt *f* and ISP) groups. The lower end of the cpc range for subIV aligns with the 2700 cpc determined in *Thermosynechococcus elongatus* (hereafter *T*. *elongatus*) by Rexroth et al. (2014) who also reported an abundance of 2500 cpc for ISP, in agreement with the known stoichiometry within the complex. However, the PSII:cyt*b*_6_*f* ratio of 1.08–1.14 reported by Fujita and Murakami (1987) alongside our determination of PSII at 24,000–44,000 cpc suggests that, in *Synechocystis*, cyt*b*_6_*f* may actually align with the higher abundance cyt*b*_6_ and ISP at 14,000–28,000 cpc.

The ISP identified in this analysis was the principal PetC1 isoform encoded by the sll1316 gene. The alternative isoform (PetC2, slr1185-encoded) which is produced under low oxygen (Summerfield et. al 2008) or high light stress (Tsunoyama et al. 2009) was undetectable. The third ISP, PetC3 (sll1182-encoded) was identified in our analyses (Supplementary Data Sets S1 and S2), however, it is localized to the cytoplasmic, not thylakoid membrane (Aldridge et al. 2008) where its function appears unrelated to photosynthetic electron transport (Veit et al. 2016a).

The minor subunits, PetG, PetL, PetM and PetN, encoded in *Synechocystis* by smr0010, ssl3803, smr0003 and sml0004 respectively (Schneider et al. 2007) were not identified in our analyses; these proteins are all smaller than 40 residues, hydrophobic and form a single transmembrane helix (TMH). Therefore, their resistance to trypsin digestion and consequent non-detection in these analyses might be expected. The putative regulator of electron transport, PetP (Ssr2998), previously characterized in *T. elongatus* (Rexroth et al. 2014; Veit et al. 2016b), was quantified at 3350–12,000 cpc (Fig. 1b). The lower end of this range coincides with the 3300 cpc determined for PetP in *T. elongatus* by Rexroth et al. (2014).

Plastocyanin (PC, PetE), the copper-containing electron carrier between cyt*b*_6_*f* and PSI is found to be highly abundant at 101,500–182,000 cpc (Fig. 1b). The alternative electron carrier to PC, cyt*c*_6_ (PetJ) which is produced in response to growth conditions with low copper levels (García-Cañas et al. 2021) is detected at a low level (Supplementary Data Sets S1 and S2) but, with representation by a single peptide, was not validated for quantification. The dominance of PC over cyt*c*_6_ is predictable under the nutrient-rich culture conditions used here; in the presence of Cu, expression of *petJ* is repressed while *petE* expression is induced by a mechanism involving the protease Slr0241 (García-Cañas et al. 2021), not detected here.

#### Photosystem I

PSI receives electrons from either PC or cyt*c*_6_ (see above) and uses solar energy to drive the reduction of Fd, which functions in a wide range of metabolic pathways including NADPH generation in a reaction catalysed by ferredoxin-NADP^+^ reductase (FNR, see below). NADPH is subsequently involved in, among other processes, the CBB cycle for CO_2_ fixation (Lea-Smith et al. 2016; Figs. 2, 3 and 5).

Four PSI subunits were quantified: PsaA–C and PsaF. Figure 1a shows abundance levels that cover the majority of data-points over the 86,000–118,500 cpc range. As in the case of the PSII D1 and D2 subunits, the data-point overlap for PsaA, PsaC and PsaF approximately fits a one subunit per complex stoichiometry (Çoruh et al. 2021). PsaB, appearing to be underestimated at 38,000–61,500 cpc, exemplifies the potential for inaccuracy with MS-based protein quantification resulting from, for example, idiosyncratic proteolysis and/or peptide ionization properties, as detailed in Supplementary Table S1. Our quantification of PSI aligns, at its lower end, with the 63,000–99,000 and 96,000 cpc determined spectrophotometrically by Fujita and Murakami (1987) and Keren et al. (2004) respectively. The latter authors also quoted their measurement of Chl content at 1.4 × 10^7^ molecules/cell. On the basis that each PSI complex houses 95 Chl molecules (Malavath et al. 2018), 96,000 cpc would account for 9.1 × 10^6^ Chls, approximately 65% of the total cellular content. Assuming the same proportion, our determination of 1.57 ± 0.04 × 10^7^ Chl molecules/cell (Supplementary Table S4) correlates with 107,500 cpc of PSI, which is within the range determined by our MS-based quantification.

#### Photosynthetic NAD(P)H dehydrogenase-like complex type-1

Under conditions of environmental stress that require an increased ATP:NADPH ratio, for example to meet a higher demand for protein synthesis, the proton gradient across the TM, utilized by the ATP synthase complex, can be elevated by cyclic electron transfer (CET; Kramer et al. 2004). One of the CET mechanisms used by cyanobacteria is mediated by photosynthetic NAD(P)H dehydrogenase-like complex type-1 (NDH-1; reviewed by Laughlin et al. 2020; Fig. 1b). In *Synechocystis*, NDH-1 is composed of 19 subunits, distributed between membrane-intrinsic and peripheral arm regions (Pan et al. 2020). In our analysis two subunits located in the latter region, NdhI and NdhK are quantified at 8550–13,500 and 7000–11,500 cpc, respectively (Fig. 1b), consistent with evidence that all NDH-1 subunits occur at one copy per complex (Pan et al. 2020).

#### ATP synthase complex

The (cyano) bacterial ATP synthase complex (Fig. 1c) comprises two main functional components, one membrane extrinsic and the other embedded in the membrane bilayer (Guo et al. 2019). The membrane-extrinsic, catalytic F_1_ component contains *α*- and *β*-subunits at three copies each with single-copy *γ*-, *δ*- and *ε*-subunits. The membrane-intrinsic, proton translocating F_O_ complex contains a single a-subunit and, in *Synechocystis*, 14 c-subunits (Pogoryelov et al. 2007). Forming the peripheral stalk connecting the two sectors are the single-copy b- and b'-subunits.

Here, we determined the ranges 67,000–83,500 cpc for α, β and 12,000–25,500 cpc for γ, δ, ε, b, b', giving a (*αβ*):(γδεbb') ratio of 2.6–7.0 (Fig. 1c). The lower end of this range reflects the known stoichiometry of the ATP synthase complex, as detailed above (Guo et al. 2019). A higher (αβ) stoichiometry, approaching 7.0, may be accommodated by our quantification of fully assembled, functional complexes together with nascent complexes composed of partially assembled modules. Indeed several F_1_ sub-assemblies have been detected in *E. coli*: γε (Rodgers and Wilce 2000), α_3_β_3_γ (Koebmann et al. 2002) and α_3_β_3_γε (Deckers-Hebestreit 2013).

In *E. coli*, ATP synthase subunits were quantified at 2700–3700 cpc for α/β and 600–1700 cpc for γ/δ/ε (Wiśniewski and Rakus 2014), giving a (αβ):(γδε) ratio of 1.6–6.2, in close agreement with the present study. Based on γ/δ/ε cpc, the cellular abundance of ATP synthase complexes in *Synechocystis* is therefore approximately 15–40-fold higher than in *E. coli.* This marked difference in ATP synthase levels between these two organisms may be predictable given their vastly divergent metabolic characteristics.

### Biosynthesis

#### Carboxysomes

Carboxysomes are 100–200 nm icosahedral structures located in the cytoplasm of cyanobacteria (Faulkner et al. 2017), each comprising a self-assembling multi-protein shell that houses two of the Calvin–Benson–Bassham (CBB) cycle enzymes: carbonic anhydrase (CcaA) and ribulose-1,5-bisphosphate carboxylase/oxygenase (RuBisCO). Two types of carboxysomes (*α* and *β*) have been defined based on their associated RuBisCO isoforms, with the *β*-type occurring in *Synechocystis* (Badger et al. 2002). The shell contains pores that selectively allow HCO_3_^−^, the substrate for CcaA to enter while preventing the exit of CO_2_ and the entry of O_2_ (Cai et al. 2009). Thus, carboxysomes are nano-compartments in which CO_2_ becomes concentrated and O_2_, the competitive inhibitor of RuBisCO with respect to CO_2_ is excluded.

Six of the seven proteins that comprise the carboxysomal shell are quantified in this study, together with one of the two proteins reported to function in assembly and organization (Cameron et al. 2013). The CcmK1–4 proteins first assemble as homohexamers alongside mixed stoichiometry K1–K2 and K3–K4 heterohexamers, each with a central pore. The hexamers then associate as a single-layer forming the shell facets (Faulkner et al. 2017). In terms of quantification, CcmK1–4 fall into two abundance groups with K1 and K2 at 79,000–169,000 and 61,000–94,500 cpc respectively and, at 10–20-fold lower abundance, K3 and K4 at 2900–8100 and 9400–19,500 cpc (Fig. 2a). These groupings align with earlier observations of K1/2 as major and K3/4 as minor shell proteins (Yeates et al. 2011). CcmL proteins, quantified here at 3550–8850 cpc, are homopentameric and form the carboxysomal vertices (Tanaka et al. 2008). Using the dimensions presented by Faulkner et al. (2017), based on TEM and cryo-EM imaging and assuming regular icosahedral geometry with 20 facets and 12 vertices, we estimate that our quantification of CcmK1 and CcmL would give 11–23 and 59–148 carboxysomes per cell respectively. The EM-based carboxysome count is six on average (Reinhold et al. 1991) therefore the excess shell protein levels are likely due to a substantial population of incomplete carboxysomes, as described in models for both biogenesis (Cameron et al. 2013) and degradation (Hill et al. 2020).

Like CcmK1–4, the shell protein CcmP oligomerizes around a central pore. However, CcmP is more complex than the single-layer CcmK1–4 since it integrates into the shell as a stacked dimer of trimers. This arrangement not only encloses an internal compartment but also provides a mechanism for gating the central pore (Cai et al. 2013). Assuming six carboxysomes per cell, alongside our quantification at 930–1900 cpc (Fig. 2a), there would be 25–50 of these CcmP gated pores per carboxysome.

Deletion mutants of the seventh shell protein CcmO have confirmed its absolute requirement for carboxysome assembly and that it associates with CcmK1/2 during the encapsulation phase (Rae et al. 2012; Cameron et al. 2013). The hypothesis that CcmO occurs at the facet edges (Rae et al. 2012) might imply that this protein is at least moderately abundant. Unexpectedly, CcmO was not detected in either this or previous (Faulkner et al. 2017) proteomic analyses.

In addition to the outer shell proteins, carboxysomes contain CcmM and CcmN, which are involved in structural organization. CcmM occurs as two isoforms (Cot et al. 2008): (1) the full-length translation product CcmM73 is located within an inner shell where it functions as a scaffold protein interacting with CcmK1-4, CcmL, CcaA and CcmN (Long et al. 2007; Kinney et al. 2012) and (2) the truncated product of an alternative downstream initiation codon CcmM52 which crosslinks RuBisCO in paracrystalline arrays for assembly into the carboxysome interior (Cameron et al. 2013). Our quantification of CcmM at 21,000–44,500 cpc might be expected, given its dual functionality and multiple interaction partners, including possibly 50,000 RuBisCO hexadecamers (see below). However, without the ability to differentiate CcmM73 and CcmM52 by our methodology, further interpretation is not applicable. CcmN, like CcmO (see above) was not detected either here or by Faulkner et al. (2017).

#### Calvin-Benson-Bassham cycle

The enzymes belonging to the CBB cycle were selected for quantification in this study by referring to https://www.genome.jp/kegg-bin/show_pathway?syn00710 and are shown in Figs. 2b, c. The first step is the conversion of HCO_3_^−^ to CO_2_ by carbonic anhydrase (CcaA), quantified here at 2400–9200 cpc (Fig. 2b). Its catalytic unit is a homodimer and, with a *k*_cat_ = 3340 s^−1^, CcaA potentially generates 4–15 × 10^6^ CO_2_ molecules cell^−1^ s^−1^ (McGurn et al. 2016). This reaction occurs inside carboxysomes (see above) where the second step, in which RuBP is carboxylated by RuBisCO, generates two molecules of glycerate-3-P. RuBisCO is often cited as the most abundant enzyme on Earth and, accordingly the result of our quantification of its large (RbcL) and small (RbcS) subunits is 156,000–204,500 and 136,000–406,500 cpc, respectively (Fig. 2b). In the majority of photoautotrophs, including cyanobacteria, RuBisCO is hexadecameric with 8 copies each of the RbcL and RbcS subunits (Andersson et al. 2008). Therefore the number of active enzyme complexes might approach 50,000 per cell. This very high abundance would mitigate for its slow carboxylation rate, estimated at ~ 0.7 s^−1^ per active site in *S. elongatus* (Flamholz et al. 2019). The cpc levels presented here would accommodate the known 1:1 stoichiometry, although the upper extent of the RbcS range at > 400,000 cpc also suggests a two-fold excess over RbcL in vivo. Similarly, a relative LFQ proteomic analysis of *Synechocystis by* Kwon et al. (2013) reported a 1.4-fold excess of RbcS.

Following its formation by RuBisCO, glycerate-3-P exits the carboxysome and the CBB cycle continues in the cytoplasm, ultimately regenerating RuBP. Intermediates downstream of glycerate-3-P in the CBB cycle additionally feed into numerous biosynthetic and metabolic processes (reviewed by Mills et al. 2020). While kinetic parameters for cyanobacterial CBB cycle enzymes are apparently not well represented in the literature (Janasch et al. 2019), some patterns do emerge that align with the quantification results shown in Figs. 2a, b. The ATP- and NADPH-utilizing steps, catalysed by glycerate-3-P kinase (Pgk), glycerate-1,3-phosphate dehydrogenase (Gap2) and ribulose-5-phosphate (Ru5P) kinase (Prk) all occupy an intermediate abundance range of 25,000–60,000 cpc, indicated by horizontal dashed lines in Figs. 2b, c. The alternative dehydrogenase isoform Gap1 principally catalyses the reverse reaction (Koksharova et al. 1998). Its lower abundance, at 4650–6900 cpc may be expected given that Gap1 activity would be counterproductive to flux through the CBB cycle. Metabolomics-based kinetic modelling in *Synechocystis* has revealed that the steps catalysed by Pgk, Prk and another enzyme within the 25,000–60,000 cpc range, fructose-1,6/sedoheptulose-1,7-bisphosphatase (FBP/SBPase, Fbp2) are strong effector reactions in the control of flux through the CBB cycle (Janasch et al. 2019). This role of Fbp2 in flux control was corroborated in *Synechocystis* by Hing et al. (2019) who also identified transketolase (TktA), another enzyme in the same intermediate abundance range, as a key player. Therefore, Pgk, Prk, Fbp2 and TktA may all be targets of the same expression control mechanism in response to ambient growth conditions. In their analysis, Janasch et al. (2019) additionally identified RuBP, FBP and SBP as intermediates that would perturb the overall steady-state stability of the CBB cycle if their cytosolic concentrations were to increase beyond a critical level. Limiting the production of RuBP, FBP and SBP might be mediated by restricting the abundance levels of upstream enzymes. In support of this hypothesis, ribose-5-phosphate isomerase A (RpiA) and ribulose-3-phosphate epimerase (Rpe), which convert ribose-5-phosphate (R5P) and xylulose-5-phosphate (Xu5P) respectively into Ru5P, the RuBP precursor, are both quantified in a relatively low abundance range (4850–12,500 and 6550–9800 cpc respectively; Fig. 2c). Similarly, production of SBP precursors glycerone-phosphate (also named dihydroxyacetone phosphate) and erythrose-4-phosphate (E4P) may be limited by the relatively low abundance of triose phosphate isomerase (TpiA) and phosphoketolase (Xfp) at 9100–19,000 and 1200–5400 cpc, respectively.

Like Gap1/2, other CBB enzymes occur as two isozymes. The reaction catalysed by RpiA, described above, is also potentially mediated by RpiB (see KEGG link above). This apparently uncharacterized protein is not detected in this analysis, prompting the conclusion that RpiA is probably the only R5P isomerase isozyme produced by *Synechocystis* under the growth conditions used here. In the case of fructose-1,6-bisphosphate (FBP) aldolase which converts G3P + glycerone-P to FBP and E4P + glycerone-P to SBP, both Fba1 and Fba2 isozymes are quantified at widely differing ranges: 3600–17,000 and 75,500–111,500 cpc, respectively (Fig. 2c), again highlighting the dominance of one isozyme over the other. This abundance pattern is repeated with quantification of Fbp1 and Fbp2 at 980–1900 and 42,000–53,500 cpc respectively. While these isozyme abundance level differences cannot be rationalized without experiments to examine the effects of different growth conditions, there is evidence that deployment of alternative CBB cycle isozymes in *Synechocystis* provides a mechanism for acclimation to environmental CO_2_ levels that extends beyond transcriptional control (Jablonsky et al. 2016).

#### Ferredoxin-dependent processes

There are nine ferredoxins (Fds) in *Synechocystis*: Ssl0020 is the isoform that mediates electron transfer from the Fe-S centers on the PsaC subunit of PSI (Yu et al. 1995). Like PC, Fd is highly abundant: although its quantification is below the validation threshold with two quantotypic tryptic peptides, it may occur above 200,000 cpc (Fig. 3a), exceeding the abundance of PSI by factor of 2–3 (cf. Figure 1a). The 10^5^ order of magnitude for the copy number of Fd in *Synechocystis* cells has been corroborated by quantitiative immunoblotting (Moal and Lagoutte 2012). This high cellular level reflects the numerous Fd-dependent metabolic processes that exist in cyanobacteria. Of particular relevance to photosynthesis, the ferredoxin NADP^+^ reductase (FNR), which catalyses the production of NADPH for CO_2_ fixation (Cassier-Chauvat and Chauvat 2014), occurs in both full-length, and truncated (FNR_s_, with alternative initiation at M113) isoforms (Thomas et al. 2006). We quantified FNR with peptides identified from both N- and C-sides of M113 (Supplementary Data Sets S5 and S6). Although the two isoforms would, therefore, be indistinguishable in this analysis, we assume that FNR_s_ would be absent under the photoautotrophic growth conditions used here since it is only detected during heterotrophic metabolism (Thomas et al. 2006). With FNR at 20,500–30,500 cpc (Fig. 3a), the PSI:FNR ratio would be approximately 4:1, which again aligns closely with a quantitative immunoblot assay by Moal and Lagoutte (2012).

In addition to these photosynthesis-related processes, Fd is required for at least nine other metabolic processes in cyanobacteria (Lea-Smith et al. 2016). Figure 3a shows quantification results for eight of these including glutamate synthase 2 (GlsF, encoded by *gltS*) at 1900–2850 cpc. The remaining seven fall into two abundance groups shown by dashed lines in Fig. 3a. Three of the five subunits of the bidirectional hydrogenase, HoxF, HoxU and HoxH are quantified here at 740–1550, 1050–1400 and 830–1350 cpc (Fig. 3a, lower abundance group), consistent with the known subunit stoichiometry (Vignais and Billoud 2007). The flavodiiron protein (FDP), comprising Flv1 and Flv3 subunits, functions as a heterodimer (Allahverdiyeva et al. 2011). In this case, our analysis is at odds with a 1:1 stoichiometry by revealing levels of 1100–1400 cpc, in the lower abundance group for Flv1 and 4050–6100 cpc in the higher abundance group for Flv3. The observation that Flv3 is substantially more abundant than Flv1 does however align with more recent evidence of FDP activity by Flv3 oligomers in addition to Flv1/3 heterodimers (Mustila et al. 2016). Other Fd-dependent enzymes in the higher abundance group are sulfite reductase (Sir) at 3150–4300 cpc and nitrite reductase (NirA) at 3600–6300 cpc (Fig. 3a),. The enzyme that acts upstream of NirA, nitrate reductase (NarB), is not detected in this analysis despite being essential for growth in a culture medium containing nitrate as the sole nitrogen source (Baebprasert et al. 2011), as used in this study. Two subunits of the nitrate transporter complex NrtA (Sll1450) and NrtC (Sll1452) are identified (Supplementary Data Sets S1 and S2), implying the cells’ probable competency in importing nitrate from the medium. In addition, not quantified here is ferredoxin–thioredoxin reductase; the catalytic subunit FtrC is identified with two tryptic peptides, below the validation threshold for LFQ, but the second subunit, FtrV, is not detected.

#### The biosynthetic pathway for chlorophyll *a*

##### Magnesium-chelatase

The biosynthesis of Chl *a* is carried out by a series of enzymes and auxiliary proteins (Fig. 3b), starting with the magnesium-chelatase (MgCh) enzyme complex. MgCh catalyses the ATP-dependent insertion of Mg^2+^ into protoporphyrin IX, which is also the substrate for ferrochelatase (FeCh) that produces heme. Thus, the insertion of Mg^2+^ or Fe^2+^ represents a branchpoint in tetrapyrrole biosynthesis (reviewed by Bryant et al. 2020).

MgCh is associated with the cytoplasmic/stromal surface of TMs (Kopečná et al. 2015; Farmer et al. 2019), as depicted in Fig. 3b, and comprises three core subunits: (i) the AAA^+^ ATPase ChlI (Fodje et al. 2001) which provides the free energy for Mg^2+^ chelation (Reid and Hunter 2004), (ii) ChlD, an allosteric regulator (Adams and Reid 2013) that also transmits the energy released by ATP hydrolysis by ChlI (Adams et al. 2016; Farmer et al. 2019) to (iii) ChlH where the active site resides (Karger et al. 2001; Sirijovski et al. 2008; Adams et al. 2020). Quantification reveals that data-points for ChlI cover a 2400–7300 cpc range while ChlD and ChlH levels are confined to 2200–3550 and 2250–2950 cpc, respectively (Fig. 3b). On the assumption that the abundance range shown by ChlD/H represents a 1:1 molar ratio for these subunits in the active MgCh complex (Farmer et al. 2019), then the level of ChlI in relation to ChlD/H is either the same or only higher by a factor of 2–3. Given that the active MgCh probably comprises multiple copies of ChlI, based on structural evidence that ChlI associates into hexamers (Gao et al. 2020) or heptamers (Reid et al. 2003), it appears that the number of fully-assembled, active MgCh complexes may be limited by the availability of ChlI subunits to 500–1200 per cell, sufficient to produce 7–16 molecules of Mg-protoporphyrin IX (MgP_IX_) s^−1^ cell^−1^, based on a *k*_cat_ of 0.013 s^−1^ (Reid and Hunter 2004; Viney et al. 2007). Association of the auxiliary MgCh subunit Gun4 is not an absolute requirement for activity in vitro but does enhance the rate of MgP_IX_ formation by a factor of 3–10 (Larkin et al. 2003; Davison et al. 2005; Davison and Hunter 2011; Adams et al. 2016). We quantified Gun4 at 570–2050 cpc (Fig. 3b), which aligns with the 500–1200 cpc range suggested for the number of active MgCh complexes per cell. If MgCh binds Gun4 in a 1:1 ratio, the rate of MgP_IX_ generation may therefore approach 160 s^−1^cell^−1^.

##### Mg-protoporphyrin IX methyltransferase

Following the insertion of Mg^2+^, MgP_IX_ is converted to Mg-protoporphyrin IX monomethyl ester (MgPME) by Mg-protoporphyrin IX methyltransferase (ChlM). The production of MgPME occurs with *k*_*cat*_ = 57 s^−1^ (Shepherd and Hunter 2004) which, alongside our quantification of ChlM at 1750–2950 cpc (Fig. 3b), equates to 99,750–168,150 s^−1^cell^−1^ thereby exceeding the rate of MgP_IX_ production by a factor of  > 1000. This marked difference may represent a mechanism for preventing the accumulation of MgP_IX_ and therefore its potentially cytotoxic effects (Tanaka and Tanaka 2007).

##### O_2_-dependent Mg-protoporphyrin IX methyl ester cyclase

In the third step of Chl biosynthesis, the C13 methylpropionyl sidechain of MgPME is cyclized by O_2_-dependent Mg-protoporphyrin IX methyl ester cyclase to form the fifth isocyclic (E) ring of 3,8-divinyl protochlorophyllide *a* (DV-PChlide). The presence of the E ring induces a change in the absorption profile, which transforms the red color of the substrate to a green product (Chen et al. 2021). The cyclase has two isoforms in *Synechocystis*, CycI and CycII, which share 56.7% sequence identity and are encoded by sll1214 and sll1874, respectively. Consistent with the normal culture aeration used here, we identified CycI at 1150–1700 cpc (Fig. 3b) as the only cyclase isoform present. CycII, which is synthesized in addition to the constitutive CycI under low O_2_ (Minamizak et al. 2008; Peter et al. 2009), was below the limit of detection in all analyses. The *k*_cat_ of 0.015 s^−1^ measured by Chen et al. (2021) for CycI is comparable to that of MgCh at 0.013 s^−1^ (determined in the absence of Gun4), giving a potential rate of MgPME to DV-PChlide conversion of 17–26 s^−1^ cell^−1^.

Like MgCh with Gun4, CycI also associates with an auxiliary protein, Ycf54, in *Synechocystis* (Hollingshead et al. 2012) and plants (Bollivar et al. 2014; Herbst et al 2018). Although the structural elements in Ycf54 that mediate its association with CycI have been characterized (Hollingshead et al. 2017), its precise role in MgPME cyclase activity is not yet defined. While our analysis shows that the Gun4 copy number appears to be in an approximate 1:1 ratio with that of assembled MgCh complexes (see above), Ycf54, at 5350–6200 cpc (Fig. 4b) is 3–fivefold more abundant than CycI. This higher stoichiometry may reflect evidence that, although CycI is membrane-associated (Tottey et al. 2003; Rzeznicka et al. 2005; Allen et al. 2008; Hollingshead et al. 2012), Ycf54 is located in both soluble and membrane fractions (Hollingshead et al. 2012).

##### Protochlorophyllide oxidoreductase (POR)

Following DV-PChlide formation, the next two steps in the Chl biosynthesis pathway result in the reduction of the C17 = C18 and C8-vinyl double bonds. In cyanobacteria, POR exists as two structurally unrelated versions: light-dependent (LPOR) and light-independent or dark-operative (DPOR) (Reinbothe et al. 2010). The latter is composed of three subunits: ChlN, ChlB, and ChlL, and none of these was detectable in our analyses (Supplementary Data Sets S1 and S2). This finding is expected for the growth conditions used here since DPOR activity is inhibited by O_2_ at > 3% and the expression of its subunits is induced only under anaerobic conditions (Yamazaki et al. 2006). LPOR is a single subunit enzyme that uses the energy from a photon absorbed by its substrate, DV-PChlide, to acquire H (with two electrons) from NADPH and a proton from a conserved Tyr residue (Heyes et al. 2006). The analysis presented in Fig. 3b reveals an abundance range of 3450–4700 cpc. A *k*_*cat*_ = 0.027 s^−1^ (Zhang et al. 2021) gives potential production rates for DV-Chlide of 93–127 s^−1^ cell^−1^, thereby exceeding the upstream cyclase step by a factor of 4–7. As suggested for the MgP_IX_ to MgPME conversion, the LPOR substrate DV-PChlide is potentially hydrogenated in the light to form DV-Chlide faster than it can accumulate.

##### 8-Vinyl reductase

In the next reduction step, catalysed by DV-(P)Chlide 8-vinyl reductase (DVR), the C8 vinyl group of DV-Chlide is converted to ethyl, producing (monovinyl) chlorophyllide (Chlide). DVR, encoded by slr1923 in *Synechocystis* (Islam et al. 2008; Ito et al. 2008), is detectable in both membrane and soluble fractions (Canniffe et al. 2014). Despite its wide distribution, DVR was quantified at the lowest abundance of all the Chl biosynthesis-associated proteins at < 500 cpc (Fig. 3b). To our knowledge, there are as yet no published steady state activity measurements for cyanobacterial DVR, therefore, the potential cellular rate of DV-Chlide to Chlide conversion cannot currently be estimated.

##### Geranylgeranyl reductase and chlorophyll *a* synthase

The final two steps in Chl biosynthesis can occur in either order (Soll et al. 1983; Proctor et al. 2022a). Geranylgeranyl reductase (ChlP) catalyses the hydrogenation of three C = C double bonds on the C_20_ isoprenoid geranylgeranyl pyrophosphate (GGPP) to produce phytyl pyrophosphate. The phytyl group is then attached via an ester linkage to the C17 propionate sidechain on Chlide by Chl *a* synthase (ChlG). Alternatively, ChlG can first attach a geranylgeranyl group to Chlide for subsequent reduction to phytyl by ChlP. ChlP is probably active on the cytosplasmic surface of the TM, whereas ChlG is predicted to be membrane-intrinsic (Gaubier et al. 1995) with up to nine TMHs (Proctor et al. 2022a). ChlP was quantified at 1150–1850 cpc, the sixth Chl biosynthesis pathway component to fall into the shared 1150–3550 cpc range indicated by horizontal dashed lines in Fig. 3b. ChlG was revealed as a member of the lower abundance group of Chl synthesis pathway components, falling in between CycI and DVR, at 450–1000 cpc (Fig. 3b). Since, to our knowledge, there are currently no published *k*_cat_ measurements for either ChlP or ChlG, their potential cellular catalysis rates remain undetermined.

#### The carotenoid biosynthesis pathway

Carotenoids are membrane-intrinsic isoprenoids synthesized by all oxygenic photoautotrophic organisms, where they play roles in the function, assembly and stability of complexes including PSII (Umena et al. 2011), PSI (Jordan et al. 2001), cyt*b*_6_*f* (Malone et al. 2019) and NDH-1 (Schuller et al. 2019). Recently, structures of some of these complexes from *Synechocystis* have been determined, revealing the positions of the carotenoids (Malavath et al. 2018; Gisriel et al. 2022; Proctor et al. 2022b). Carotenoids are also essential for photoprotection, quenching ROS-generating Chl triplet states and superoxide (Cogdell et al. 2000), and in cyanobacteria the photoactive orange carotenoid protein (OCP) is involved in thermal dissipation of excess energy from the phycobilisome antenna (Muzzopappa and Kirilovsky 2020).

Carotenoid biosynthesis (summarized in Fig. 3c and reviewed by Canniffe and Hitchcock (2021)) commences with the condensation of the products of the 2-C-methyl-D-erythritol 4-phosphate (MEP) pathway isopentenyl pyrophosphate (IPP) and dimethylallyl pyrophosphate (DMAP) producing geranyl pyrophosphate (GPP). In oxygenic phototrophs this reaction, together with two subsequent additions of IPP to GPP to produce GGPP, are catalysed by the GGPP synthase (CrtE). Subsequently, two molecules of GGPP are condensed to generate 15-*cis*-phytoene by phytoene synthase (CrtB), which then undergoes a series of desaturations and isomerizations resulting in the production of all-*trans*-lycopene, the common precursor of the major carotenoid species utilized by *Synechocystis*, namely β-carotene, myxoxanthophyll, echinenone, zeaxanthin and synechoxanthin (Lagarde and Vermaas 1999; Graham et al. 2008).

In our analysis, CrtE, which is also required for Chl biosynthesis, is quantified, along with phytoene desaturase (CrtP), ζ-carotene desaturase (CrtQ) and the prolycopene isomerase CRT-ISO (CrtH), all of which occur early in the pathway in the synthesis of all-*trans*-lycopene. Figure 3c shows that CrtE, CrtP and CrtH occur at abundance levels in the 380–1100 cpc range (horizontal dashed lines), while CrtQ is higher at 1300–1900 cpc. In addition to CrtE, CrtP, CrtQ and CrtH, the other enzymes required to generate all-*trans*-lycopene are CrtB and ζ-carotene isomerase (Z-ISO). A *Synechocystis crtB* mutant cannot synthesize carotenoids, is light sensitive and lacks functional PSII (Sozer et al. 2010), suggesting the enzyme is likely to be present just below the detection limit. Given that the cells used here were grown under constant illumination, the known photo-lability of the 15-*cis* bond of 9,15,9′-tri-*cis*-ζ-carotene (Li et al. 2007) may explain non-detection of the *Synechocystis* Z-ISO (Slr1599; Proctor et al. 2022c), although in plants Z-ISO is important in both “dark” and light-exposed tissues (Chen et al. 2010). Our detection of CrtH, the other carotenoid isomerase, suggests photoisomerization alone is insufficient in the case of the second carotenoid isomerization step; a S*ynechocystis crtH* mutant produces normal carotenoids under light conditions due to photoisomerization of the *cis*-bonds in prolycopene, albeit at different ratios to the wild-type organism (Masamoto et al. 2001).

The remaining eight enzymes are either not synthesized under the culture conditions used here or occur at < 200–500 cpc. The major lycopene cyclase CruA (Xiong et al. 2017) is identified but at levels below the threshold for quantification (Supplementary Data Sets S1 and S2), whereas CruP, the lycopene cyclase function of which is controversial (Maresca et al. 2007; Liang et al. 2008), is not detected. Similarly, CruF and CruG, specific to myxoxanthophyll biosynthesis (Graham and Bryant 2009), CruE and CruH, required for synthesis of synechoxanthin (Graham and Bryant 2008), and CrtO, the ketolase for echinenone and 3-hydroxy-echinenone biosynthesis (Fernández-González et al. 1997) are not detected, nor is CrtR, which adds the hydroxyl groups to the *β*-rings of zeaxanthin, myxoxanthophyll and 3-hydroxy-echinenone (Lagarde and Vermaas 1999).

Levels below the threshold of identification for carotenoid biosynthesis enzymes were also apparent in two previous proteomic studies that employed sub-cellular fractionation to potentially enhance proteomic coverage (Xu et al. 2021; Baers et al. 2019). This low copy number may be a consequence of the low turnover of carotenoids under moderate illumination conditions and/or extremely efficient enzymes meaning high cellular levels are not required.

#### The phycobilin biosynthesis pathway

Bilins, linear tetrapyrroles derived from heme, are light-harvesting chromophores covalently attached to phycobiliproteins, which assemble to form the phycobilisome antenna complex (Dominguez-Martin et al. 2022). The biosynthesis pathway of bilins, reviewed by Bryant et al. (2020), is common with that of Chls up to protoporphyrin IX, where it diverges from the branch initiated by the insertion of Fe^2+^ catalysed by ferrochelatase (FeCh, HemH) to produce heme. Heme oxygenase (Hox) then cleaves the heme macrocycle to produce the linear molecule biliverdin IXα, which is subsequently converted to a bilin via a Fd-dependent bilin reductase; *Synechocystis* produces only phycocyanobilin (PCB). We quantify FeCh (Slr0839; 1300–1600 cpc) and HoxI (PbsA1, 1800–3800 cpc), while the PCB-ferredoxin oxidoreductase (PcyA) is detected but present at < 500 cpc. As expected, Hox2 (PbsA2), which is produced under microoxic conditions (Yilmaz et al. 2010), is not identified.

The attachment of phycobilins to specific cysteine residues of phycobiliproteins requires specific bilin lyases (Scheer and Zhao 2008). In cyanobacteria such as *Synechocystis* and *Synechococcus* sp. PCC 7002, three PCBs are attached to a phycocyanin heterodimer, one to CpcA by a heterodimeric lysase comprised of CpcE and CpcF (Fairchild et al. 1992), one to CpcB by a CpcS/CpcU family lyase (Saunée et al. 2008) and one to CpcB by a CpcT family lyase (Shen et al. 2006). CpcS/CpcU also attach PCB to the core allophycocyanin antenna subunits ApcA, ApcB, ApcD and ApcF (Zhao et al. 2007). We quantify CpsS1 (CpcU, 2300–3800 cpc) and CpcT (2700–3700 cpc) but not CpcE, CpeF or CpcS2 (CpcS), suggesting these are present at < 500 cpc.

An alternative fate of biliverdin Ixα is reduction to bilirubin by the pyridine nucleotide-dependent biliverdin reductase (BvdR); the *Synechocystis* enzyme is quantifed by only 1–2 peptides at < 500 cpc (Supplementary Data Set S4). Although not a direct component of the phycobiliosme, bilirubin is suggested to act as a ROS scavenger (Hayes and Mantle 2009) and BvdR is important for normal phycobiliprotein biosynthesis in *Synechocystis* (Schluchter and Glazer 1997).

### Photosystem assembly and repair

#### Coordination of chlorophyll and photosystem II biosynthesis

##### ChlG and HliD

As stated above, the step in the Chl biosynthesis pathway in which either a geranylgeranyl or phytyl chain is ester-linked to the Chlide macrocycle is catalysed by ChlG. Immunoprecipitation (IP) experiments employing FLAG-tagged ChlG have shown that this membrane-intrinsic enzyme co-isolates with the single-TMH proteins HliC (Niedzwiedzki et al. 2016) and HliD (Chidgey et al. 2014), two of the four high light-inducible proteins (Hlips) present in *Synechocystis* (HliA-D; Komenda and Sobotka 2012). The ChlG-HliC/D complex also incorporates Chl and carotenoids (Chidgey et al. 2014; Niedzwiedzki et al. 2016), implicating HliC and HliD in photoprotection specifically during PSII, but not PSI (see below), assembly in which the delivery of Chl is coordinated with the co-translational insertion of nascent apoproteins into the membrane (Chidgey et al. 2014; Knoppová et al. 2014). According to our analyses, the abundance of ChlG is 450–1000 cpc (Fig. 3b) with HliD 3–sevenfold higher at 2000–3200 cpc (Fig. 4a). HliC was also identified in this study (Supplementary Data Sets S1 and S2) however, recovery of its single proteotypic tryptic peptide from artificial SIL standard proteins proved non-reproducible during initial tests (results not shown) and LFQ would be below the validation threshold with < 3 peptides. The greater abundance of HliD over ChlG is supported by the observation that carotenoids only bind to HliC/D dimers, not monomers (Shukla et al. 2018), implying that the functional units of these Hlips are dimers. The detection of ChlG- and HliD-containing complexes at > 100 kDa by native-PAGE/immunoblot analysis (Proctor et al. 2020) further suggests that PSII assembly centers may comprise multiple copies of ChlG and HliC/D dimers.

##### Ycf39

An additional protein co-isolating with Flag-ChlG in IP analysis is Ycf39 (Slr0399; Chidgey et al. 2014), although its interaction with the complex is lost under high-light conditions (Proctor et al. 2018). Ycf39 is predicted to be hydrophilic and its interaction with the membrane-intrinsic ChlG-HliC/D complex is on the TM cytoplasmic surface (Knoppová et al., 2014). IP analysis using FLAG-tagged Ycf39 showed that its direct binding partner is dimeric HliC/D (Staleva et al., 2015) and native-PAGE/immunoblot analysis confirmed the association of Ycf39 with early intermediates in the PSII assembly pathway (Knoppová et al., 2014, 2022; Heinz et al. 2016; Konert et al. 2022). Ycf39, is quantified here at 1200–1800 cpc (Fig. 4a), therefore HliD alone outnumbers Ycf39 by a factor of two, highlighting the possibility that all copies of Ycf39 are bound to Hlip dimers.

##### YidC and SecY

The identification of ChlG-HliC/D-Ycf39 complexes highlights the PSII assembly process in terms of Chl delivery and photoprotection. The additional detection of the membrane insertase YidC in FLAG-ChlG IP analyses (Chidgey et al. 2014; Niedzwiedzki et al. 2016) establishes the direct link with co-translational integration of PSII apoproteins into the TM. While the ChlG-HliC/D-Ycf39 complex is evidently specific to PSII assembly (Knoppová et al. 2014, 2022; Pascual-Aznar et al. 2021), YidC participates in the co-translational insertion of a wide range of membrane-intrinsic proteins (Kudva et al. 2013). YidC was quantified here at 1000–3350 cpc (Fig. 4a), coincident with the 2500–3000 cpc determined in *E. coli* (Urbanus et al. 2002; Kudva et al. 2013). SecY, the only subunit of the SecYEG translocon identified here, is quantified at 110–830 cpc. Again, this range is in close agreement with the 200–600 cpc in *E. coli* (Kudva et al. 2013). The perhaps unexpectedly low abundance of these proteins in *Synechocystis* may be rationalized on the basis that a chaperone-type function is similar to catalysis in that, after membrane insertion of the substrate protein, YidC and SecYEG are released and available to bind a new substrate. Furthermore, a recent fluorescent imaging study of mRNA sequences mapping to PsaA and PsbA has revealed that translation sites for these membrane-integral PS subunits are not widely distributed but instead confined to the interior cytosol-facing surface of the TMs (Mahbub et al. 2020). TM-intrinsic protein insertion appears therefore to be localized and the levels of YidC and SecYEG may reflect this.

#### Thylakoid membrane biogenesis and photosystem II assembly

##### CurT

The characteristic morphology of thylakoids in cyanobacteria is dependent on CurT, an integral membrane protein that induces membrane curvature (Heinz et al. 2016). Accordingly, inactivation of *curT* results in the development of aberrant TM structure and additionally a 50% reduction in PSII accumulation compared to wild-type. This effect on PSII levels is accompanied by the elimination of the biogenesis centers, more recently referred to as ‘convergence zones’ (Rast et al. 2019), implicating CurT in the formation of these features as part of normal TM morphology (Heinz et al. 2016). Quantification of CurT in this study indicates the possibility of cellular levels approaching 140,000 cpc (Fig. 4a). Imaging by both immunofluorescence and immunogold labelling supports this finding since CurT is detectable, not only in association with convergence zones but also throughout the TM on both concave and convex surfaces (Heinz et al. 2016).

##### PratA, Pitt and Slr0151

Specifically localized to the convergence zones where they function as PSII assembly factors are three tetratricopeptide repeat (TPR) proteins: PratA (Slr2048; Klinkert et al. 2004), Pitt (Slr1644; Schottkowski et al. 2009) and Slr0151 (Rast et al. 2016). Using high-resolution cryo-electron tomography, it has been demonstrated that, within the convergence zones, membranes continuous with the TM are in close contact with the CM (Rast et al. 2019). This juxtaposition enables a mechanism whereby PratA delivers Mn^2+^ from the periplasm to the membrane-integrated PsbA precursor (pD1; Stengel et al. 2012). The level of PratA was quantified here at 320-630 cpc (Fig. 4b).

Pitt (encoded by slr1644) is anchored in the TM via an N-terminal TMH and inactivation of slr1644 was shown to reduce the accumulation of LPOR (see above) by 70% (Schottkowski et al. 2009). These authors suggested that the association of LPOR with the TM might be via its binding to Pitt. With representation by only two tryptic peptides, Pitt was not validated for quantification. However, iBAQ abundance scores are consistent with a level of Pitt at < 500 cpc (Supplementary Data Set S4). Our quantification of LPOR at 3450–4700 cpc (see above), up to tenfold more abundant than Pitt, would align with the further idea of an LPOR-Pitt membrane-attachment complex that directs a relatively small LPOR sub-population to the convergence zone for an, as yet unknown function.

Slr0151 has been characterized as functioning in PSII biogenesis (Rast et al. 2016) and repair (Yang et al. 2014) and slr0151 inactivation results in impaired TM morphology (Rast et al. 2016). Unlike the other two TPR proteins PratA and Pitt, and probably consistent with its wider distribution both within convergence zones and throughout the TMs (Rast et al., 2016), Slr0151 is quantified in our analysis at 4450–7900 cpc (Fig. 4b). Furthermore, there is evidence that Slr0151 has a greater range of interaction partners than PratA and Pitt including PSII subunits PsbA/D1 and PsbC/CP43 (Yang et al. 2014), and is involved in the modulation of Fd-5 phosphorylation (Angeleri et al. 2018).

##### Pam68, Ycf48 and RubA

The Pam68 assembly factor, first characterized in Arabidopsis as TM-intrinsic, was identified by homology with Sll0933 in *Synechocystis* (Armbruster et al. 2010). Using FLAG-tagged Pam68, Bučinská et al. (2018) co-isolated a complex containing PsbB/CP47 as the only PSII subunit represented, alongside YidC, SecY and several riboproteins. These associations reveal that the probable function of Pam68 is facilitating the co-translational insertion of PsbB/CP47 into the TM and possibly also the correct apoprotein configuration for the delivery of Chl (Bučinská et al. 2018). Co-isolating with Pam68 in IP analysis is the lumenal protein Ycf48 (Rengstl et al. 2013; Bučinská et al. 2018). Ycf48 is homologous with Arabidopsis HCF136 (Meurer et al. 1998) and was shown to be essential for the accumulation of PsbB/CP47 and PsbC/CP43 in the TM (Rengstl et al. 2013). It has been proposed that Ycf48 may, like Pam68, facilitate co-translational Chl delivery to nascent apoproteins (Crawford et al. 2016). We quantified Pam68 and Ycf48 at 1250–1600 and 3600–5150 cpc, respectively (Fig. 4b). We suggest that Ycf48 is approximately threefold more abundant than Pam68 because it associates with a greater number of precursor modules: CP47, CP43 (Rengstl et al. 2013), D1 and RCII (Yu et al. 2018). Another component of the D1 assembly complex, with evidence of a direct association with Ycf48, is RubA (Slr2033; Kiss et al. 2019), quantified here at 2850–7150 cpc.

##### Psb27 and Psb28

Analysis of intermediates in PSII biogenesis by immunoblotting identified Psb27 as a component of assembly modules containing PsbC/CP43 (Komenda et al. 2012; Fig. 4b) and recent structural analyses by cryo-EM revealed not only docking sites on PsbC/CP43 (Zabret et al. 2021) but also the induction of conformational changes in PsbB/CP47 and PsbD/D2 (Huang et al. 2021). According to IP analysis, Psb28 co-isolates with the RC47 assembly module (Bečková et al. 2017), docking with both PsbA/D1 and PsbD/D2 subunits, where it induces temporary conformational changes that may protect nascent PSII from photo-damage until the Mn_4_CaO_5_ cluster is assembled and water oxidation activated (Zabret et al. 2021). Psb27 and Psb28 (Sll1398) were quantified as relatively high abundance assembly factors at 3500–60,500 and 28,000–100,000 cpc respectively, reflecting their participation in interactions with multiple PSII assembly intermediates (Komenda et al. 2012; Bečková et al. 2017; Pascual-Aznar et al. 2021). A second isoform of Psb28 encoded by slr1739 (Boehm et al. 2012a) and proposed to have a divergent function (Bečková et al. 2017) was identified with only two peptides in the label-free DDA analysis (Supplementary Data Set S4) and therefore not validated for quantification.

#### Thylakoid membrane biogenesis and photosystem I assembly

It may be argued that CurT facilitates the accumulation of PSII indirectly via its role in inducing the correct TM architecture and that this activity of CurT also provides the TM environment for PSI biogenesis. Unexpectedly however, inactivation of *curT* has been shown to have no effect on PSI abundance (Heinz et al. 2016), highlighting a link between TM biogenesis and PSI assembly via an alternative mechanism.

##### VIPP1

In addition, referred to as IM30, VIPP1 was first identified in chloroplasts as essential for TM biogenesis (Kroll et al. 2001), with membrane insertion of PsaA and PsaB compromised in a Δ*vipp1* strain of *Synechococcus* sp. PCC 7002. This evidence suggests that VIPP1 is actually functional in PSI biogenesis and that the presence of PSI, with CurT, is required for the formation of normal TM architecture (Zhang et al. 2014). Structural characterization has established that VIPP1 binds to the membrane surface as homo-oligomers of > 1 MDa (Aseeva et al. 2004) forming stacked rings that induce curvature of the membrane into a central hydrophobic channel (Gupta et al. 2021). In view of this MDa size, the 40,500–45,000 cpc abundance determined in this analysis (Fig. 4c) suggests the actual number of functional VIPP1 units within the TM may be only 1000–1200. Unlike CurT, which may be maintained at high abundance because its membrane curvature activity is a constant requirement over the entire TM, VIPP1 is proposed to have a localized function at convergence zones where it may participate in lipid transfer between the CM and nascent TM (Gupta et al. 2021).

##### Ycf3, Ycf37 and Ycf4

Although PSI assembly occurs so rapidly that the isolation of intermediate subcomplexes has proved challenging (Schöttler et al. 2011), several assembly factors have emerged from the investigation of mutants with defective PSI accumulation (Wilde et al. 1995; Boudreau et al. 1997; Bartsevich and Pakrasi 1997; Wilde et al. 2001).

Ycf3 is a hydrophilic TPR protein that was found to co-isolate in IP analysis with PsaA and PsaD on the cytoplasmic surface of the TM (Naver et al. 2001). A similar strategy demonstrated the association of subunits PsaA-D with Ycf37, a TPR protein anchored in the TM by one TMH, also on the cytoplasmic surface. Furthermore, Ycf37 co-migrated with PSI(1), not PSI(3) after sucrose gradient centrifugation, indicating its possible role in PSI trimerization (Dühring et al. 2006). Ycf3 and Ycf37 are quantified here at comparable abundance levels: 2950–4750 and 1750–5300 cpc respectively (Fig. 4c), suggesting that these proteins may have similar stoichiometric relationships with nascent PSI complexes. Their approximate 20-fold lower abundance than the cellular PSI population (86,000–118,500 cpc; Fig. 1a) supports the view that Ycf3 and Ycf37 operate as chaperones, interacting only transiently with their respective binding partners.

The use of TAP-tagged Ycf4 has revealed interactions with six subunits: PsaA-PsaF, with stability dependent on bound PsaF (Ozawa et al. 2009). In a model proposed by Nellaepalli et al. (2021), Ycf3 is the first to bind to a newly synthesized PsaA/B heterodimer, followed by Ycf4 which stabilizes the complex as more subunits join, followed by Ycf37. Ycf4 is quantified at 6800–14,000 cpc (Fig. 4c), almost twofold higher than Ycf3 and Ycf37. This 1:1:2 stoichiometry for Ycf3:Ycf37:Ycf4 may be explained if Ycf4 is active as a dimer.

#### FtsH proteases

The PsbA/D1 subunit of PSII is highly susceptible to photo-oxidative damage as part of its normal function (Adir et al. 2003). To maintain continuity of PSII activity and enable acclimation to changing illumination, a repair mechanism has evolved in oxygenic phototrophs (Nixon et al. 2005). Four homologous, membrane intrinsic, ATP-dependent Zn-metalloproteases encoded by *ftsH1-4* are involved in PSII repair and a wide range of other cellular processes. Their functional diversity is based on the assembly of different combinations of both homo- and hetero-oligomeric complexes (Mann et al. 2000; Boehm et al. 2012b). All four were quantified in the analysis reported here, ensuring that only unique proteotypic tryptic peptides were used. The *Synechocystis* FtsH proteases all have similar sequence identities (41–47%) to the single FtsH occurring in *E. coli*, with a reported abundance of 660 cpc (Wiśniewskia and Rakus, 2014). This level is in close agreement with our determination of FtsH1 at < 1000 cpc (Fig. 4d), suggesting that FtsH1 is the basic isoform (Bittner et al. 2017). The 3–sixfold higher levels of FtsH2, FtsH3 and FtsH4 at 1800–5150, 2400–3000 and 2800–5950 cpc, respectively, may reflect functions that extend beyond the remit of a basic FtsH protease, with a greater number of protein targets. Both FtsH2 and FtsH3 are functional in the degradation of photo-damaged PsbA/D1 (Silva et al. 2003; Komenda et al. 2006), specifically as an FtsH2/3 complex (Boehm et al. 2012b). An FtsH1/3 complex plays a role in the regulation of gene expression in acclimation to nutrient stress (Krynická et al. 2014; 2019).

## Conclusions

We employed four mass spectrometry-based quantification methods, which revealed the cellular levels of 97 proteins involved in photosynthesis, the biosynthesis of carotenoid, chlorophyll and bilin pigments, membrane assembly, the light reactions of photosynthesis, fixation of carbon dioxide and nitrogen, hydrogen, and sulfur metabolism. Figure 5 summarizes the cellular locations and associations of these proteins, also indicating that regulatory and biosynthetic components are generally less abundant than those involved in bioenergetic reactions. We also found disparities in the abundances of subunits relative to their stoichiometries apparent in high-resolution structures, as expected given that both complete complexes and assembly intermediates would contribute to the analysis. Furthermore, we were able to calculate cellular levels for some large complexes such as photosystems, assemblies such as carboxysomes and VIPP1 oligomers, and hexameric FtsH Zn-metalloproteinases.

Our quantitative proteomic baseline for wild-type *Synechocystis* enhances our understanding of this model organism and will be a valuable resource for the photosynthesis community, as well as forming the basis for synthetic biology projects aimed at manipulating biosynthetic, metabolic and energy-transducing pathways. *Synechocystis* is an important model organism for engineering photosynthetic metabolism and knowing the numbers of the essential components will inform attempts to use this bacterium as a chassis for using sunlight, CO_2_ and water to produce valuable metabolites and increased biomass. While the focus of this study has been on the 97 proteins described, cellular levels of 1081 proteins are also presented.

## Supplementary Information

Below is the link to the electronic supplementary material.

Supplementary file1 (XLSX 141 KB)

Supplementary file2 (XLSX 141 KB)

Supplementary file3 (XLSX 30 KB)

Supplementary file4 (XLSX 53 KB)

Supplementary file5 (XLSX 70 KB)

Supplementary file6 (XLSX 60 KB)

Supplementary file7 (XLSX 435 KB)

Supplementary file8 (DOCX 3409 KB)

## Data Availability

The mass spectrometry proteomics data have been deposited to the ProteomeXchange Consortium (http://proteomecentral.proteomexchange.org) via the PRIDE partner repository (Perez-Riverol et al. 2019) with the dataset identifier PXD035632. Other information can be provided by the corresponding author on reasonable request.
