# Supplementary material for: Absolute quantification of cellular levels of photosynthesis-related proteins in Synechocystis sp. PCC 6803
Source: Photosynth Res. 2022 Dec 21;155(3):219–45. doi: 10.1007/s11120-022-00990-z (PMC9958174; doi:10.1007/s11120-022-00990-z)
Supplement: Supplementary file 8 — Supplementary file8 (DOCX 3409 KB) [file 11120_2022_990_MOESM8_ESM.docx]

**Absolute quantification of cellular levels of photosynthesis-related proteins in *Synechocystis* sp. PCC 6803**Philip J. Jackson^1,2,^, Andrew Hitchcock^1^, Amanda A. Brindley^1^, Mark J. Dickman^2^ and C. Neil Hunter^1,^*

^1^Plants, Photosynthesis and Soil, School of Biosciences, University of Sheffield, UK
^2^Department of Chemical and Biological Engineering, University of Sheffield, Sheffield, UK
*Author for correspondence: ([p.j.jackson@sheffield.ac.uk](mailto:p.j.jackson@sheffield.ac.uk))

**Supplementary results and discussion**

**Cell count validation**

To enable cpc quantification, the three replicate *Synechocystis* cultures, with absorption at 750 nm of 0.6-0.7, were sampled for cell counting by hemocytometer immediately before harvesting. Counts were 8.24 ± 1.4 x 10^7^ cells mL^-1^, equating to 1.18-1.37 x 10^8^ cells mL^-1^ AU^-1^, comparable to the 1.45 x 10^8^ cells mL^-1^ AU^-1^ determined by Lea-Smith et al. (2014) using an automated counter. After lysis and solubilization, whole cell extracts containing the equivalent of 1.6 x 10^8^ cells were used as the starting point for further sample processing. In the SIL-DDA method, extracts were spiked with known amounts of the artificial SIL protein standards at this point. Therefore quantification using ^14^N:^15^N tryptic peptide ion intensity ratios could be directly related to the initial 1.6 x 10^8^ cells. In the LFQ methods, cell numbers were correlated with the protein content of the cell extracts, measured as described in Supplementary Fig. S1. This approach was validated by the close agreement between our estimate of cellular protein content at 2350 fg cell^-1^ and previously published values of 2600 fg cell^-1^ (Touloupakis et al. 2015) and 1804-2516 fg cell^-1^ (Zavrel et al. 2017). Supplementary Fig. S1 further shows strong correlation (LFQ-DDA: R = 0.96 and LFQ-DIA: R = 0.98) between our calculated cell numbers and C-phycocyanin β-subunit ITG-AAR peptide ion intensities as indicators of sample loading in the MS analyses.

**Protein identifications**

Nine photosystem (PS) subunits, five cyt*b*_6_*f* subunits, plastocyanin (PC), two photosynthetic NAD(P)H dehydrogenase-like complex type-1 (NDH-1) subunits, eight ATP synthase subunits, sixteen Calvin-Benson-Bassham (CBB) cycle enzymes, seven carboxysomal proteins, ferredoxin (Fd), nine Fd- dependent enzymes, eleven proteins involved in Chl biosynthesis, four enzymes in each of the carotenoid and phycobilin biosynthesis pathways and twenty proteins involved in TM biogenesis/PS assembly were quantified in terms of as copies per cell (cpc) by MS. Since all MS-based absolute quantification methods have potential vulnerabilities that may compromise accuracy, alongside their advantages, we employed four alternative methods: one calibrated with SIL standards and three LFQ methods calibrated with a commercial protein standard mixture (UPS2). These methods are described in Supplementary Table S1. In the SIL and two of the LFQ methods the mass spectrometer was programmed for DDA, automatically selecting tryptic peptide ions to generate product ion spectra. In the fourth method, DIA, product ion spectra were generated by the sequential selection of subsections of the mass-to-charge ratio (*m/z*) range without targeting specific ions. Database searches of the SIL-DDA mass spectra were performed using MaxQuant software which discounts the contribution by the ^15^N-labeled artificial standard proteins. The number of proteins identified in the nine data sets (three technical repeats of three biological replicates) was 1341-1374, representing 38.1-39.2% of the theoretical *Synechocystis* proteome (Supplementary Data Set S1). Inter-replicate correlation coefficients for the protein-level total ion intensities are 0.941-0.976 (Supplementary Table S2). Searches of the LFQ-DDA mass spectra identified 1315-1334 *Synechocystis* proteins (Supplementary Data Set S2), representing 37.5-38.0% of the theoretical proteome. Since protein identification in the LFQ-DIA analyses was achieved using a spectral library derived from the LFQ-DDA search results, a specific LFQ-DIA results data set would be redundant here. Inter-replicate correlation coefficients for the *Synechocystis* proteins in LFQ-DDA are similar at 0.970-0.983 (Supplementary Table S3). The slightly lower protein identification numbers for the LFQ-DDA analyses are the probable consequence of the additional 42.4 ng of calibration standards per MS analysis, which would have a greater negative effect on the number of *Synechocystis*-derived peptide ions selected for product ion scans than the 2.3 ng of ^15^N-labelled standards spiked into each SIL-DDA analysis.

In the SIL-DDA analyses, phycobiliproteins contributed 51-56% of the total *Synechocystis* protein intensity, derived from the intra-analysis summed tryptic peptide ion counts (Supplementary Data Set S1) and 45-49% (excluding standards) in LFQ-DDA (Supplementary Data Set S2). This proportion in terms of ion counts is expected given the very high abundance of phycobilisomes in *Synechocystis* when grown in nutrient-rich media (Anderson and Toole 1998), as in this study. The potentially problematic dominance of phycobiliproteins in the proteome, which could lower the number of other protein identifications, has been frequently addressed in many comparative quantitative proteomic analyses of *Synechocystis* by fractionation of cell extracts. For example, recent studies employing rate-zone centrifugation (Baers et al., 2019) and a combination of size exclusion and ion exchange chromatography (Xu et al., 2021) identified 2445 (70% proteomic coverage) and 2906 (83% proteomic coverage) proteins respectively. To eliminate the potential for errors in absolute quantification introduced by the addition of upstream subcellular or protein/peptide fractionation steps, as in a previous study of *E. coli* (Wiśniewskia and Rakus 2014), we analysed total protein extracts from detergent-solubilized *Synechocystis* cell pellets. Although this approach would also include damaged/senescent cells and covalent modifications that might contribute anomalous protein abundance levels, the effects of noncovalent ligands and membrane localization should be minimized. We present the absolute quantification of proteins spanning four orders of magnitude, representing the complete abundance range from <1000 to >100,000 cpc of 97 proteins representing the complexes and pathways that are the focus of this study.

**Absolute quantification method survey**

As shown in Supplementary Data Sets S3-S6, the median coefficients of variation (CVs) for protein abundance determinations using the SIL-DDA, iBAQ-DDA, Top3-DDA and Top3-DIA methods are 13.1%, 11.0%, 12.9% and 10.0% respectively. These metrics of precision are both similar to each other and in agreement with previous validation studies (Venable et al. 2004; Silva et al 2006: Pratt et al. 2006; Schwanhäusser et al. 2011; Muntel at al. 2014; Millán-Oropeza et al. 2022). In addition, relatively high CVs (>50%) are a feature of a minority of data distributions found in both this and the aforementioned studies. While recognizing the potential vulnerabilities of quantification by SIL-DDA (Supplementary Table S1), the accuracy of our analysis was optimized by manual curation of spectral peak assignments and followed previously published strategies for proteotypic peptide selection (Supplementary Data Set S3; Brownridge et al. 2013; Qian et al. 2013). In common with the other LFQ studies is our use of the commercially available recombinant human protein standard mixture (UPS2) as calibrant to enable the derivation of protein molar amounts from peptide ion intensities. To confirm the potential accuracy of our analyses, Supplementary Data Sets S4-S6 show correlation coefficients of 0.89-0.99 (*p* < 0.0001) spanning 1000-500,000 cpc. Although calibration in this way is essential for MS-based label-free absolute quantification, a comparison between UPS2-calibrated Top3-DIA and SIL/synthetic peptide-based (Kirkpatrick et al. 2005; see above) absolute quantification protocols listed proteins that ranged from closely matching to differing by two orders of magnitude (Muntel et al. 2014). Therefore, our requirement for low-cost, large scale approaches is a compromise in terms of accuracy for at least some of our target proteins. Although we limited our focus to 97 quantifiable proteins, we show abundances of 1081 proteins in Supplementary Data Set S7.

Supplementary Figs. S2-S5 show boxplots of validated cellular levels of the 97 proteins, determined by between one and all four MS-based quantification methods. In the SIL-DDA analyses, all quantypic peptides that gave validated cpc values (Supplementary Data Set S3) are represented by individual boxplots in magenta. In cases where two or more methods/SIL-peptides provide cpc values, it is apparent that the data-points for any given protein fit into different method/SIL-peptide distributions. For statistical validity the data-points generated by the different methods cannot be condensed into a single central tendency ± variation metric for each target protein. Instead, the distributions shown by the boxplots in Supplementary Figs. S2-S5 are merged to provide probable consensus cpc ranges for each target protein that account for either (a) the interquartile data-points within >2 coincident distributions with the exclusion of outlier distributions or (b) all interquartile data-points where none of the distributions are coincident. The consensus cpc ranges are shown as bars in Figs. 1-4.

**Table S1** Summary of the mass spectrometry-based protein quantification methods used in this study.

**A** Calibrated with stable isotope labelled (SIL) artificial standard proteins expressed in *E. coli*.

| **Details** | **References** |
| --- | --- |
| Target proteins (^14^N) and artificial SIL (^15^N) standard proteins are co-digested with Lys-C + trypsin. | Pratt et al. (2006)  Brownridge et al. (2013) |
| Quantification is based on ^14^N/^15^N ion intensity ratios of quantotypic peptides. |  |
| Proteolysis rates for target proteins and SIL standard proteins may be different. | Brun et al. (2007)  Scott et al. (2015) |
| Peptides released more rapidly may be more susceptible to non-specific proteolysis and deamidation. | Lin et al. (2020)  Ren et al. (2009) |
| Anomalously low intensity of either ^14^N or ^15^N peptide will lead to depressed or inflated target protein quantification respectively. | Hammel et al. (2018) |
| Different quantotypic peptides from the same target protein may give different ^14^N/^15^N ion intensity ratios. | Brownridge et al. (2013)  Hammel et al. (2018) |

**B** Calibrated with a commercially available dynamic range protein standard mixture (UPS2).

| **Details** | **References** |
| --- | --- |
| UPS2 is digested with Lys-C + trypsin and the peptides added to the samples prior to MS analysis. | Schwanhäusser et al. (2011) |
| Intensity-based absolute quantification (iBAQ):  Peptide ion intensities are summed and normalized to the theoretical number of tryptic peptides of 6-30 residues. |  |
| iBAQ: Target proteins with higher or lower numbers of quantotypic peptides per unit MW compared with UPS2 proteins will lead to depressed or inflated target protein quantification respectively. | Peng et al. (2012)  Chang et al. (2018) |
| Top3: Ion intensities of the 3 highest intensity peptides are summed. | Silva et al. (2006) |
| iBAQ/Top3:  Validation threshold ≥3 peptides. Some target proteins have <3 quantotypic peptides. | Schwanhäusser et al. (2011)  Silva et al. (2006) |
| iBAQ/Top3:  Ionization properties of quantotypic peptides may lead to depressed or inflated target protein quantification. | Peng et al. (2012)  Chang et al. (2018) |

**Table S2** Correlation matrix of total protein ion intensities derived from the SIL-DDA analyses.

| **Biological replicate-technical replicate** | | | | | | | | | |
| --- | --- | --- | --- | --- | --- | --- | --- | --- | --- |
| **1-1** | **1-2** | **1-3** | **2-1** | **2-2** | **2-3** | **3-1** | **3-2** | **3-3** |  |
|  |  |  |  |  |  |  |  |  |  |
| **1-1** | 1.000 | 0.971 | 0.955 | 0.954 | 0.946 | 0.962 | 0.954 | 0.957 | 0.957 |
|  |  |  |  |  |  |  |  |  |  |
| **1-2** | 0.971 | 1.000 | 0.948 | 0.954 | 0.941 | 0.963 | 0.951 | 0.954 | 0.951 |
|  |  |  |  |  |  |  |  |  |  |
| **1-3** | 0.955 | 0.948 | 1.000 | 0.956 | 0.968 | 0.955 | 0.956 | 0.949 | 0.957 |
|  |  |  |  |  |  |  |  |  |  |
| **2-1** | 0.954 | 0.954 | 0.956 | 1.000 | 0.952 | 0.963 | 0.966 | 0.949 | 0.969 |
|  |  |  |  |  |  |  |  |  |  |
| **2-2** | 0.946 | 0.941 | 0.968 | 0.952 | 1.000 | 0.952 | 0.948 | 0.941 | 0.949 |
|  |  |  |  |  |  |  |  |  |  |
| **2-3** | 0.962 | 0.963 | 0.955 | 0.963 | 0.952 | 1.000 | 0.961 | 0.951 | 0.963 |
|  |  |  |  |  |  |  |  |  |  |
| **3-1** | 0.954 | 0.951 | 0.956 | 0.966 | 0.948 | 0.961 | 1.000 | 0.961 | 0.976 |
|  |  |  |  |  |  |  |  |  |  |
| **3-2** | 0.957 | 0.954 | 0.949 | 0.949 | 0.941 | 0.951 | 0.961 | 1.000 | 0.957 |
|  |  |  |  |  |  |  |  |  |  |
| **3-3** | 0.957 | 0.951 | 0.957 | 0.969 | 0.949 | 0.963 | 0.976 | 0.957 | 1.000 |
|  |  |  |  |  |  |  |  |  |  |

These analyses incorporated artificial stable isotope labelled (SIL) standard proteins with the mass spectrometer programmed for data-dependent acquisition (DDA). The data-files were processed using MaxQuant to provide total ion intensities for the identified proteins (Supplementary Data Set S1), excluding the SIL standards. Spearman rank correlation coefficients are shown.
**Table S3** Correlation matrix of total protein ion intensities derived from the LFQ-DDA analyses.

| **Biological replicate-technical replicate** | | | | | | | | | |
| --- | --- | --- | --- | --- | --- | --- | --- | --- | --- |
| **1-1** | **1-2** | **1-3** | **2-1** | **2-2** | **2-3** | **3-1** | **3-2** | **3-3** |  |
|  |  |  |  |  |  |  |  |  |  |
| **1-1** | 1.000 | 0.983 | 0.982 | 0.970 | 0.970 | 0.975 | 0.970 | 0.975 | 0.973 |
|  |  |  |  |  |  |  |  |  |  |
| **1-2** | 0.983 | 1.000 | 0.982 | 0.976 | 0.968 | 0.972 | 0.975 | 0.981 | 0.979 |
|  |  |  |  |  |  |  |  |  |  |
| **1-3** | 0.982 | 0.982 | 1.000 | 0.973 | 0.967 | 0.974 | 0.972 | 0.976 | 0.973 |
|  |  |  |  |  |  |  |  |  |  |
| **2-1** | 0.970 | 0.976 | 0.973 | 1.000 | 0.973 | 0.976 | 0.975 | 0.974 | 0.975 |
|  |  |  |  |  |  |  |  |  |  |
| **2-2** | 0.970 | 0.968 | 0.967 | 0.973 | 1.000 | 0.981 | 0.968 | 0.974 | 0.962 |
|  |  |  |  |  |  |  |  |  |  |
| **2-3** | 0.975 | 0.972 | 0.974 | 0.976 | 0.981 | 1.000 | 0.971 | 0.977 | 0.966 |
|  |  |  |  |  |  |  |  |  |  |
| **3-1** | 0.970 | 0.975 | 0.972 | 0.975 | 0.968 | 0.971 | 1.000 | 0.977 | 0.975 |
|  |  |  |  |  |  |  |  |  |  |
| **3-2** | 0.975 | 0.981 | 0.976 | 0.974 | 0.974 | 0.977 | 0.977 | 1.000 | 0.977 |
|  |  |  |  |  |  |  |  |  |  |
| **3-3** | 0.973 | 0.979 | 0.973 | 0.975 | 0.962 | 0.966 | 0.975 | 0.977 | 1.000 |
|  |  |  |  |  |  |  |  |  |  |

These label-free quantification (LFQ) analyses incorporated commercially-available standard proteins (UPS2) with the mass spectrometer programmed for data-dependent acquisition (DDA). The data-files were processed using MaxQuant to provide total ion intensities for the identified proteins (Supplementary Data Set S2). Spearman rank correlation coefficients are shown for the *Synechocystis* proteins with the UPS2 proteins excluded.
**Table S4** Chlorophyll *a* determination in the wild-type *Synechocystis* cells used in this study.

| **Calculation steps** | **Replicate** | |
| --- | --- | --- |
|  | **1** | **2** |
| A_665.2_ (methanol extract) | 0.258 | 0.247 |
| A_652.0_ (methanol extract) | 0.136 | 0.118 |
| A_750.0_ (methanol extract) | 0.038 | 0.021 |
| Corrected A_665.2_ | 0.220 | 0.226 |
| Corrected A_652.0_ | 0.098 | 0.097 |
| Corrected A_665.2_ x 16.29 | 3.58 | 3.68 |
| Corrected A_652.0_ x 8.54 | 0.837 | 0.828 |
| Chl (µg) in 1.2 x 10^8^ cells | 2.75 | 2.85 |
| Chl molecules in 1.2 x 10^8^ cells | 1.85 x 10^15^ | 1.92 x 10^15^ |
| Chl molecules per cell | 1.54 x 10^7^ | 1.60 x 10^7^ |
| Mean | 1.57 x 10^7^ | |
| SD | 0.042 x 10^7^ | |

Chlorophyll content was determined according to Porra et al. (1989) in 2 x 1 mL samples containing 1.2 x 10^8^ cells (A_750_ = 0.65) from a culture selected at random from those used in this study.


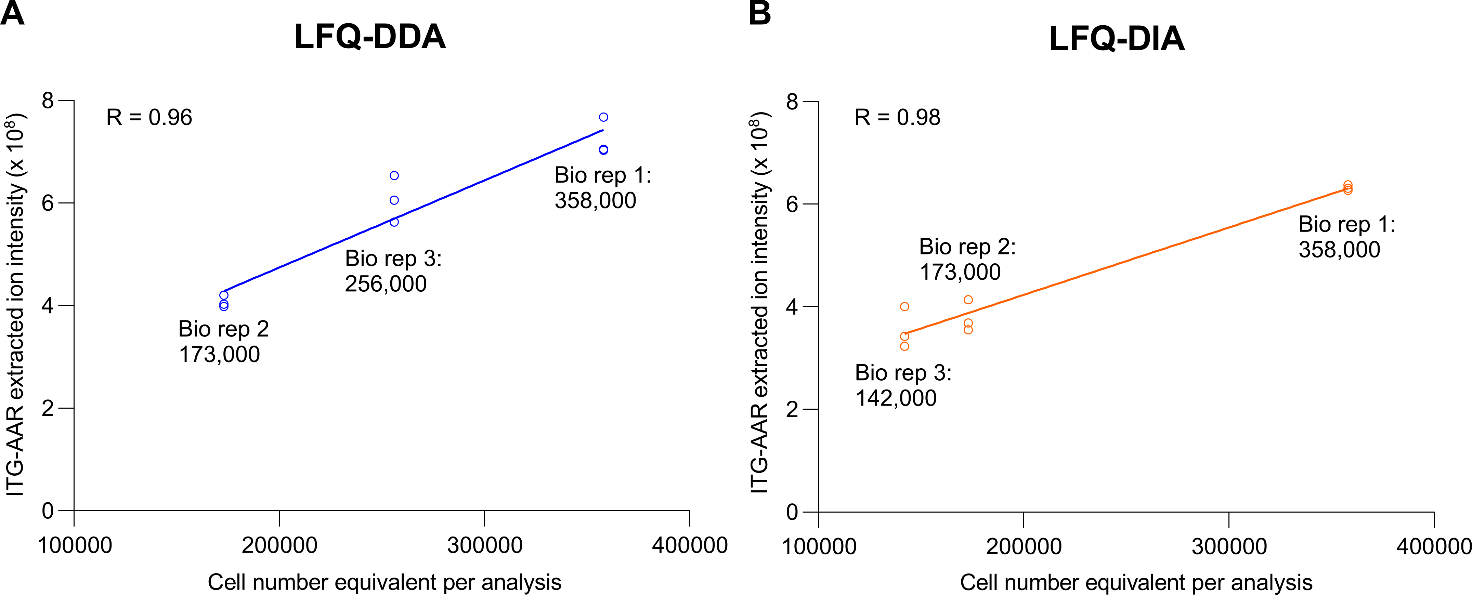


**Figure S1** Correlation of the cell number equivalents subjected to nanoLC-MS/MS analysis with C-phycocyanin β-subunit ITG-AAR peptide extracted ion intensities. *Synechocystis* cells were solubilized and the proteins extracted by precipitation as described in *Materials and methods* and by Krynická et al. (2019). Protein pellets from the three biological replicates were dissolved in 20 µL 8 M urea, 100 mM Tris-HCl pH 8.5 and protein concentration determined, after dilution with water, by Nanodrop at 280 nm to give a protein content of 2350 ± 22 fg cell^-1^. This metric was used to calculate cell equivalents per analysis from the pre-digestion protein concentrations for samples used in the LFQ-DDA and LFQ-DIA analyses. These LFQ analyses were calibrated by the introduction of commercially-available UPS2 standards, after separate trypsin/endoproteinase Lys-C digestion, immediately before nanoLC-MS/MS analysis. In contrast the SIL-DDA analyses employed ^15^N-labelled artificial standard proteins introduced before protein extraction by precipitation. Without co-processed standards, the LFQ analyses were instead validated by the correlation between the cell number equivalent per nanoLC-MS/MS analysis and C-phycocyanin β-subunit ITG-AAR peptide ion intensities as an indicator of sample loading onto the MS system, as shown. The correlation coefficients (R) are Pearson with data-points for three technical repeats of three biological replicates.

| 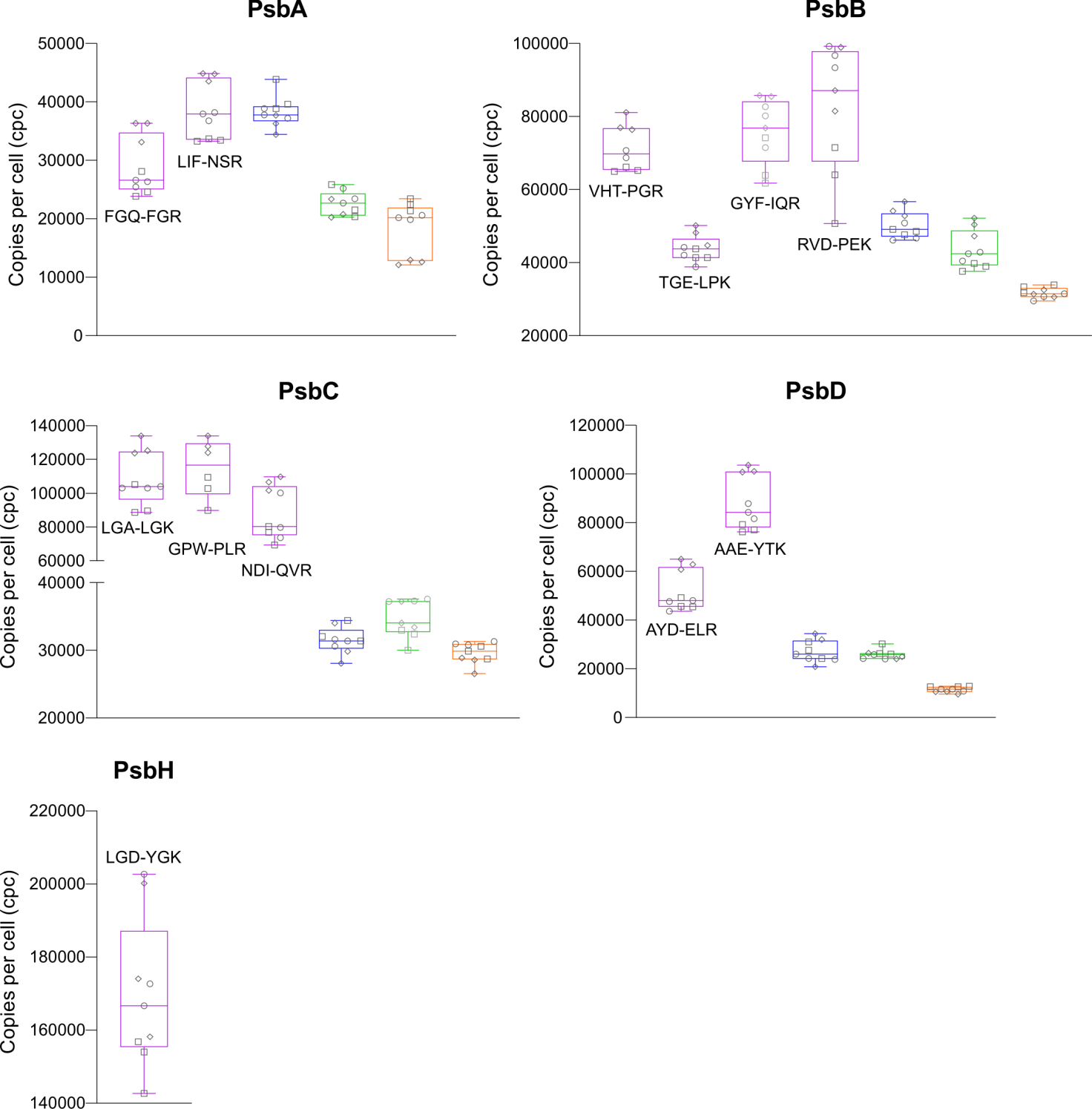 | 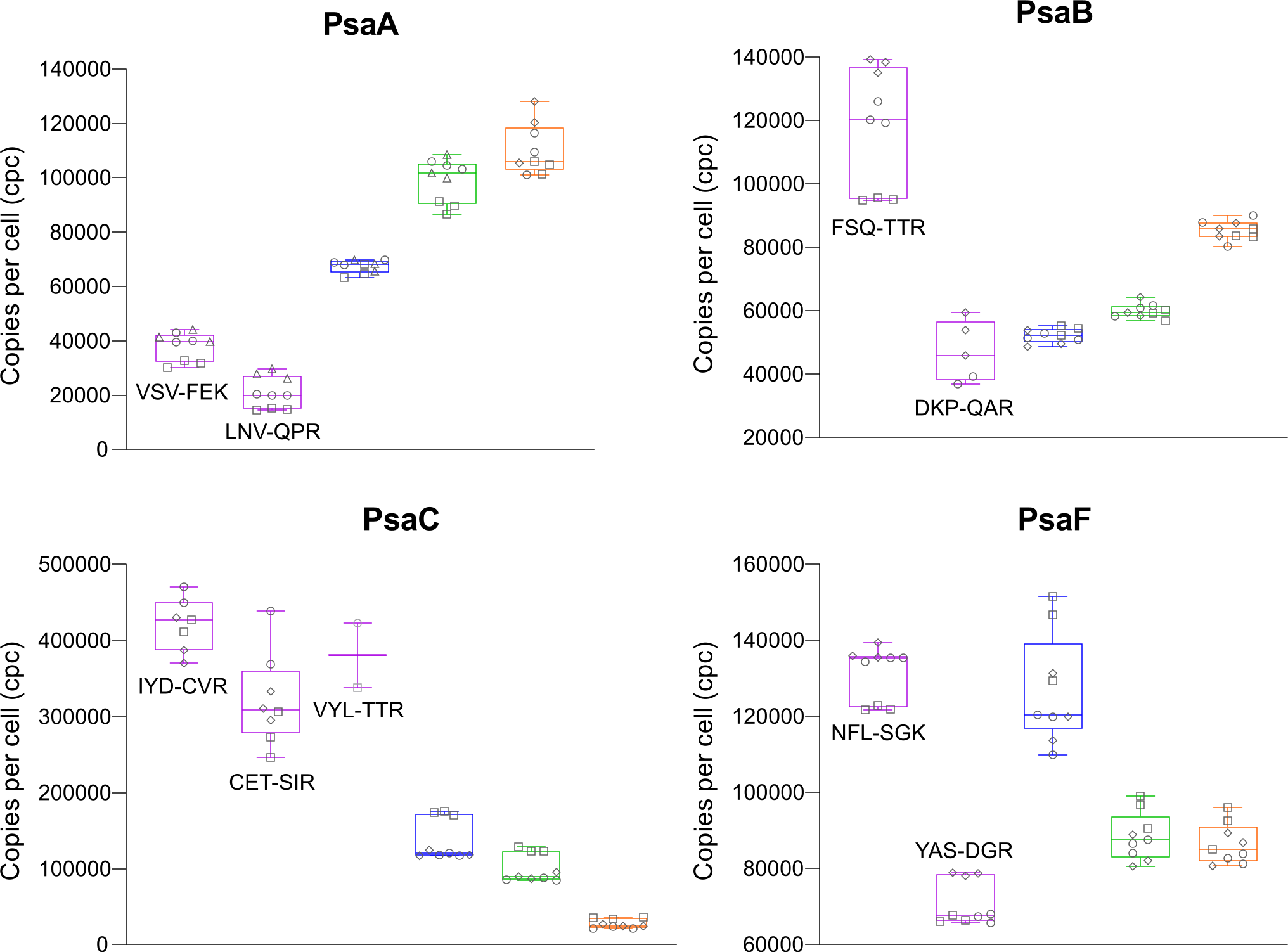 |
| --- | --- |
| 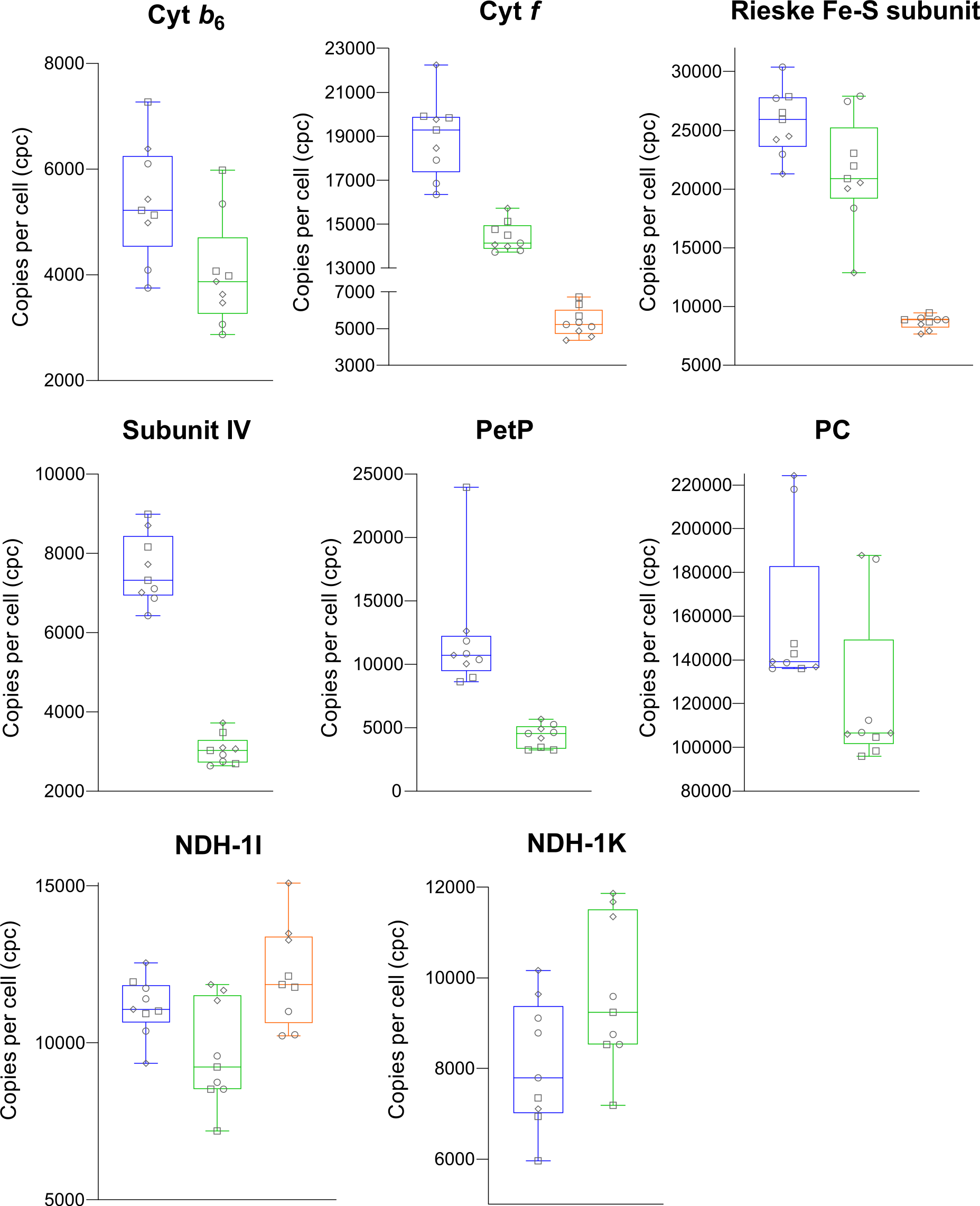 | 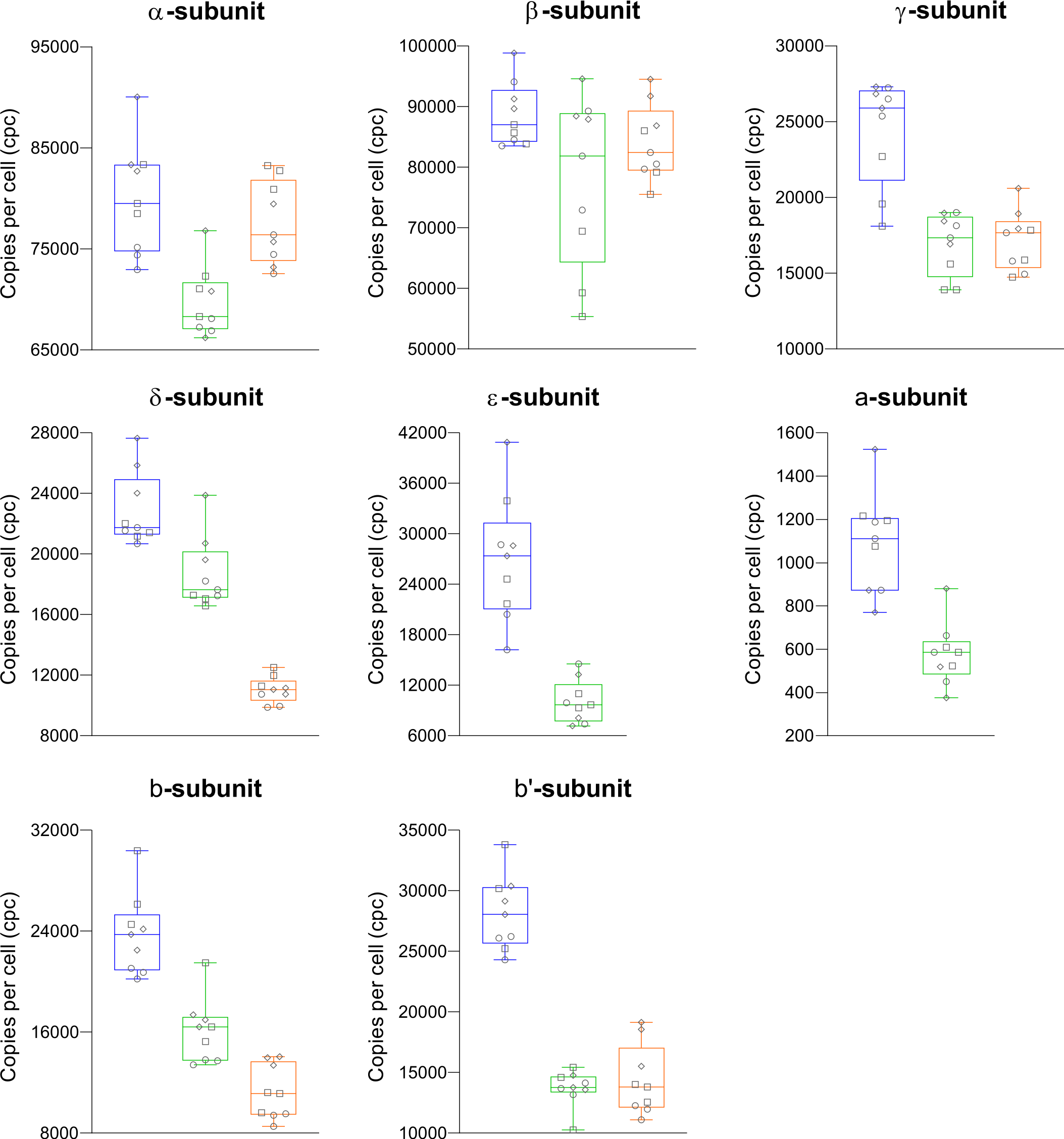 |

**Figure S2** Boxplots showing copies per cell (cpc) distributions of protein complexes involved in the conversion of solar to chemical energy. Protein names are defined in the main article figure legends. Data-points for biological replicates 1, 2 and 3 are represented by circles, squares and diamonds respectively, with 3 technical repeats of each. The boxplots, showing interquartile range, median and minimum/maximum, are colored according to the quantification methods used: SIL-DDA (magenta, with quantotypic peptides identified), iBAQ-DDA (blue), Top3-DDA (green) and Top3-DIA (orange), using the data-points tabulated in Supplementary Data Sets S3-S6 respectively.

| 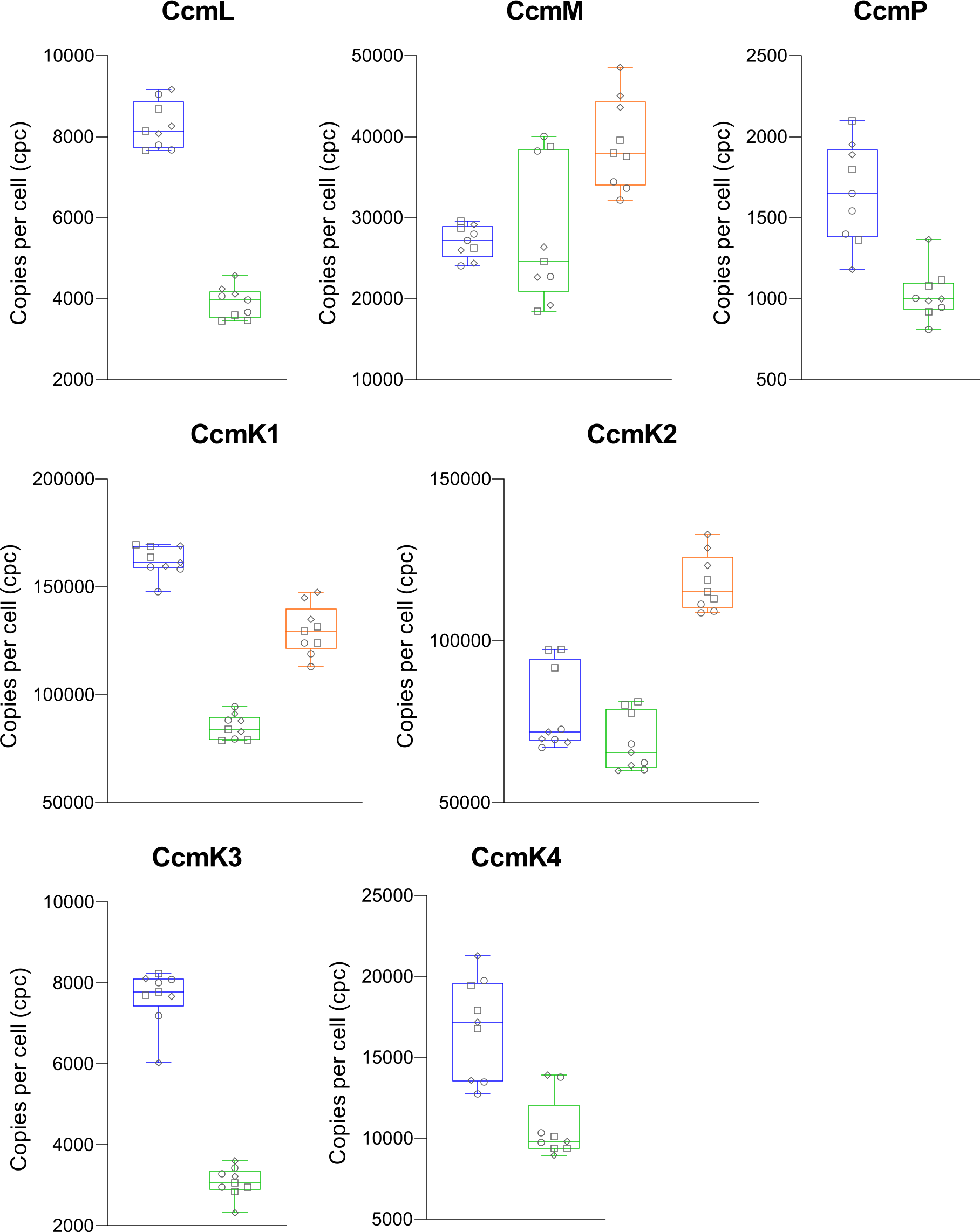 | 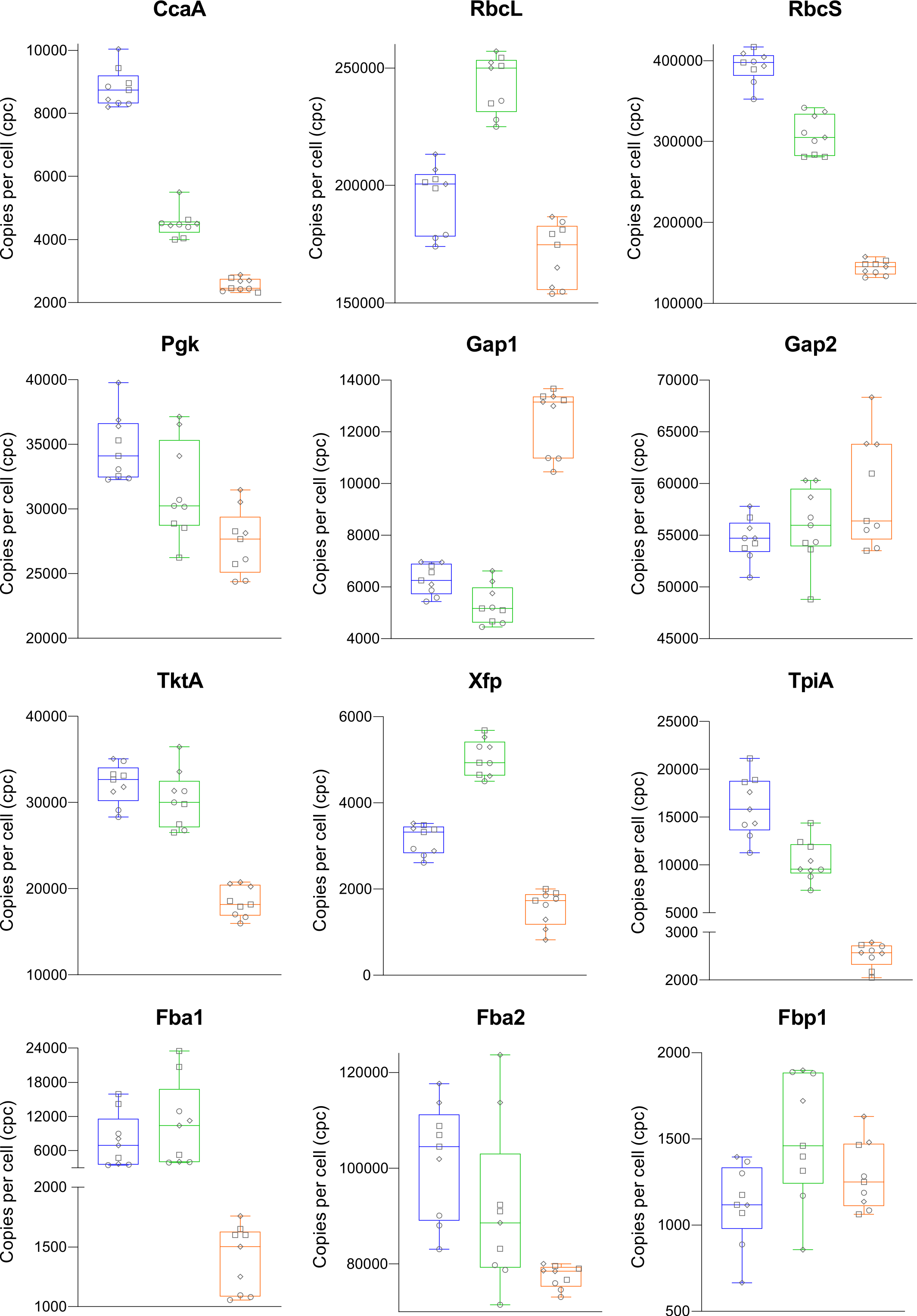 |
| --- | --- |
| 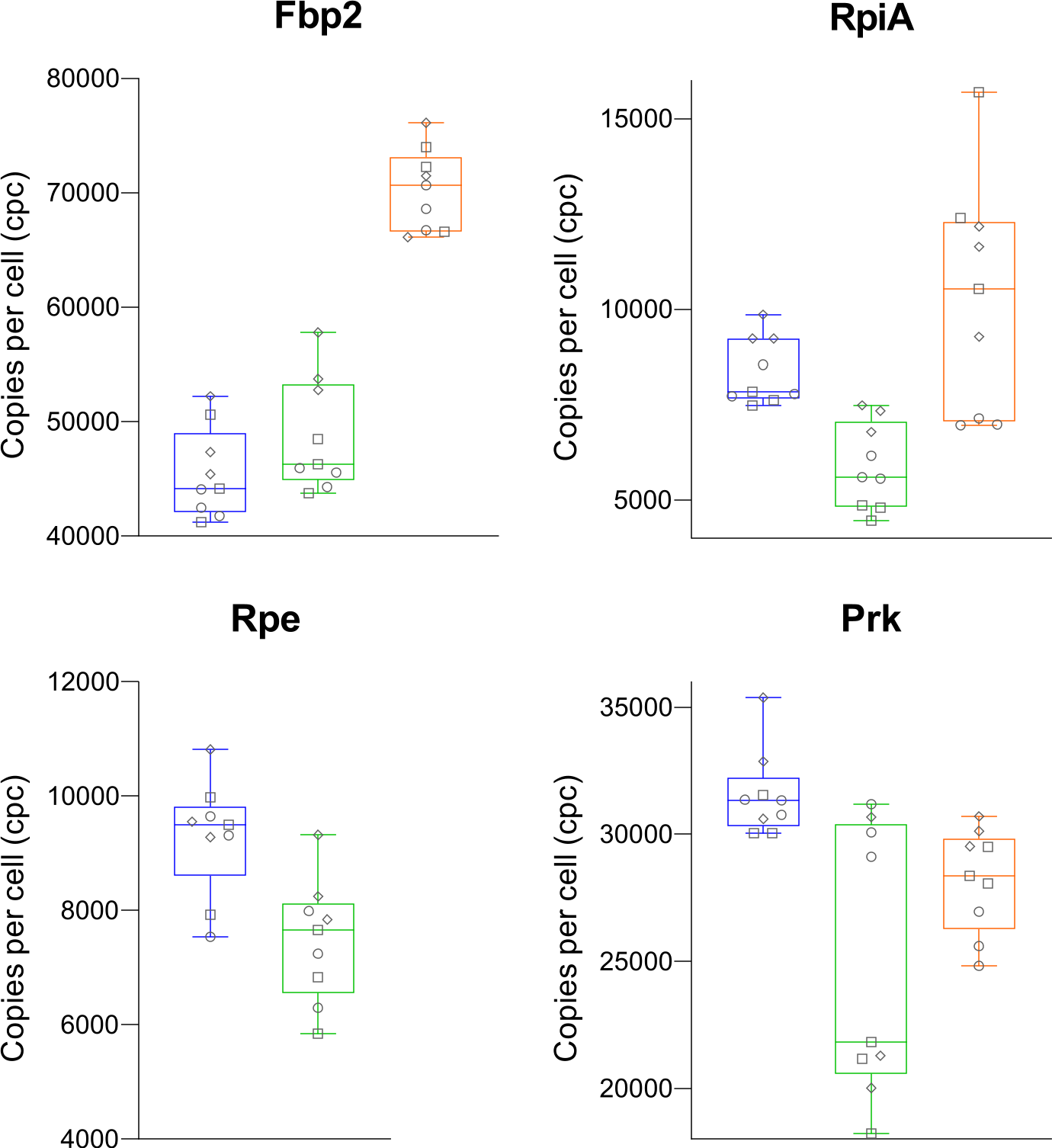 |  |

**Figure S3** Boxplots showing copies per cell (cpc) distributions of carboxysomal proteins and enzymes of the Calvin-Benson-Bassham cycle. Data-points for biological replicates 1, 2 and 3 are represented by circles, squares and diamonds respectively, with 3 technical repeats of each. The boxplots, showing interquartile range, median and minimum/maximum, are colored according to the quantification methods used: iBAQ-DDA (blue), Top3-DDA (green) and Top3-DIA (orange), using the data-points tabulated in Supplementary Data Sets S4-S6 respectively.

| 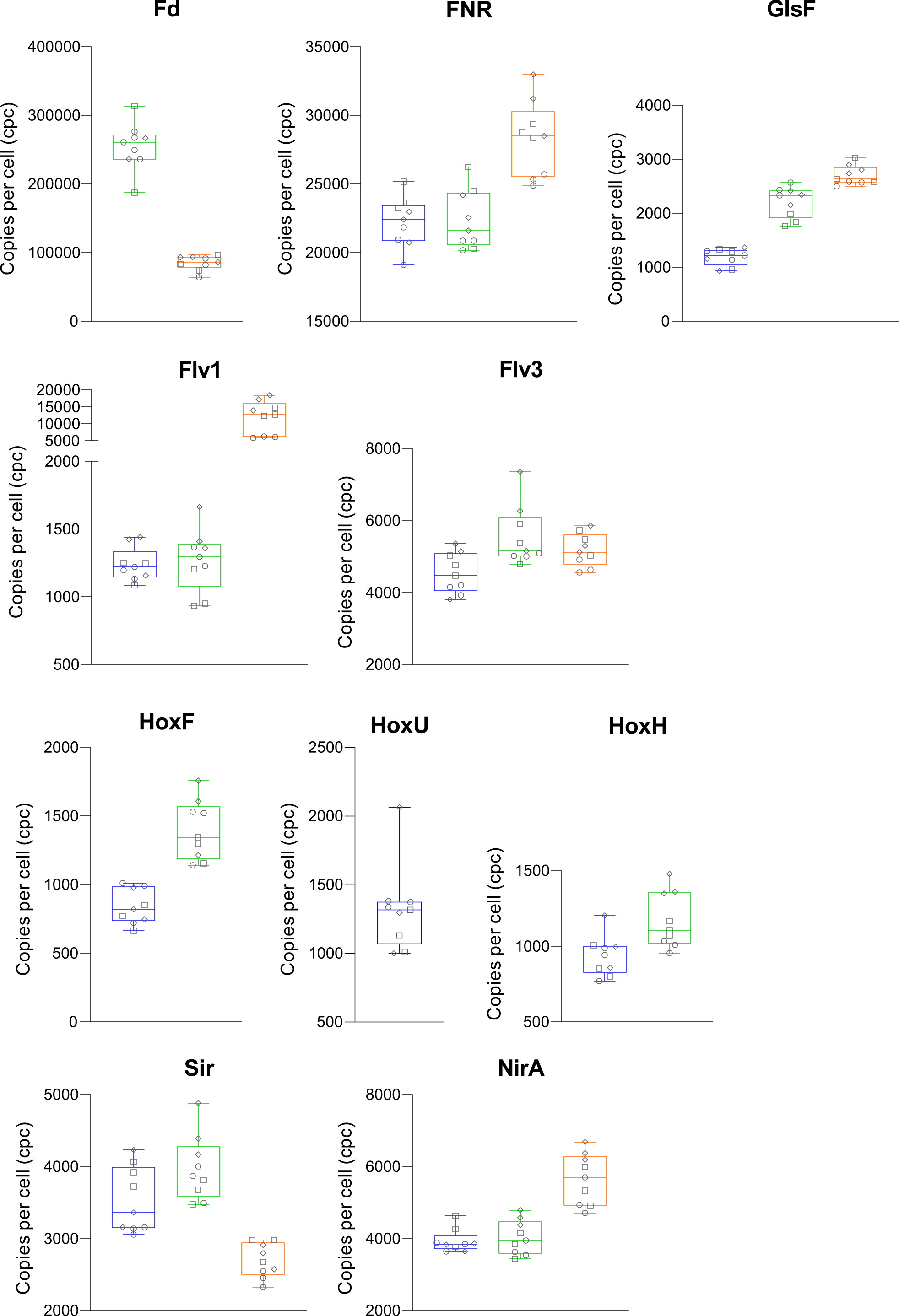 | 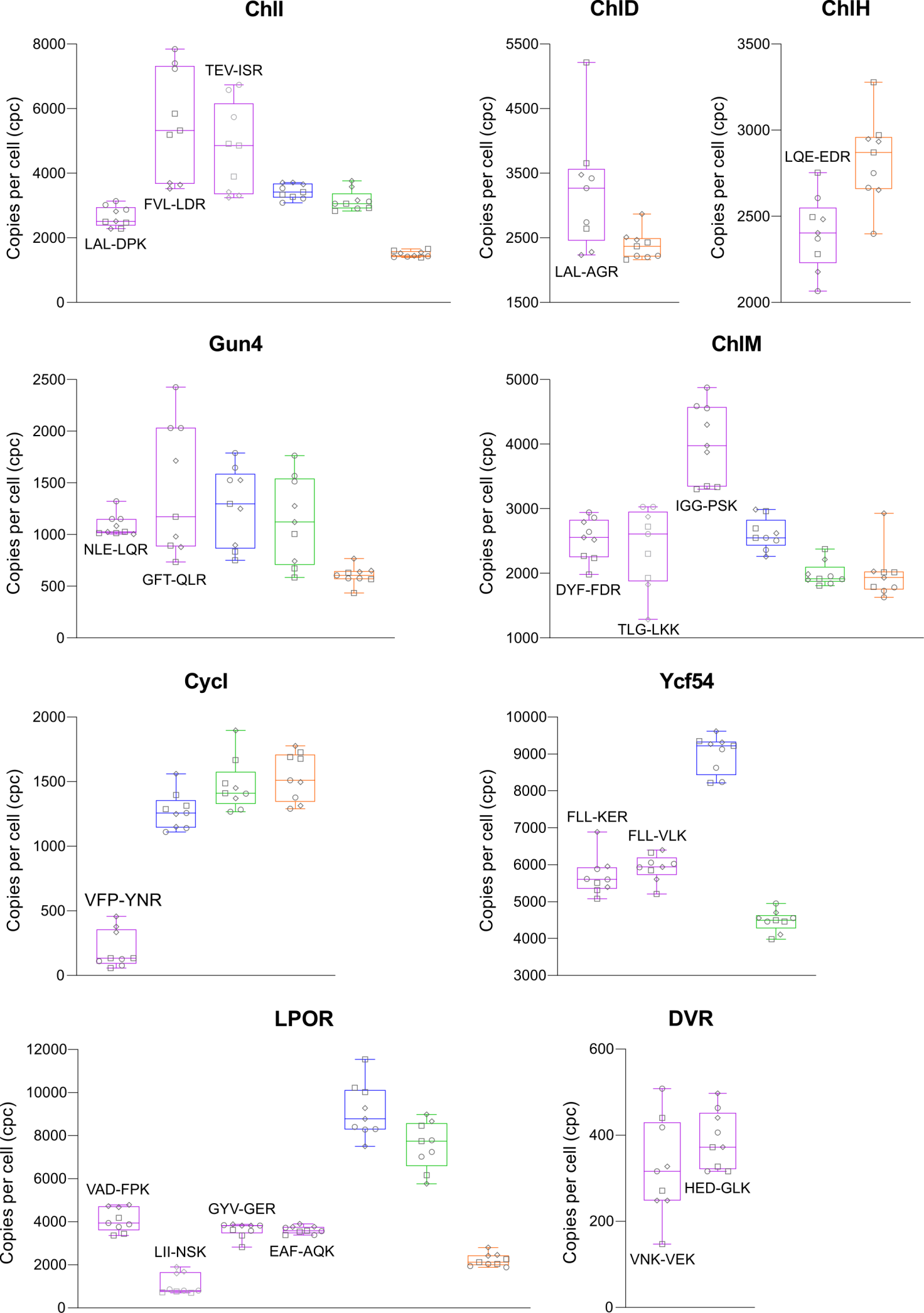 |
| --- | --- |
|  | 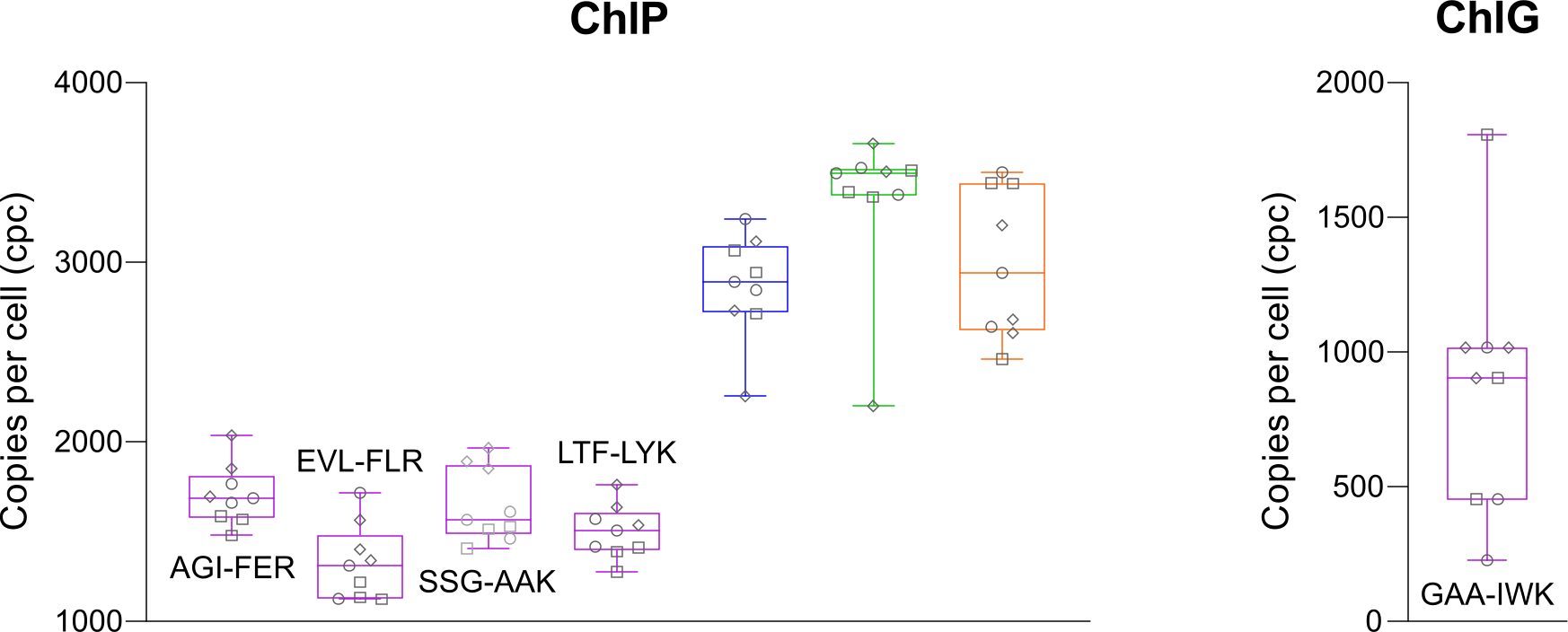 |
| 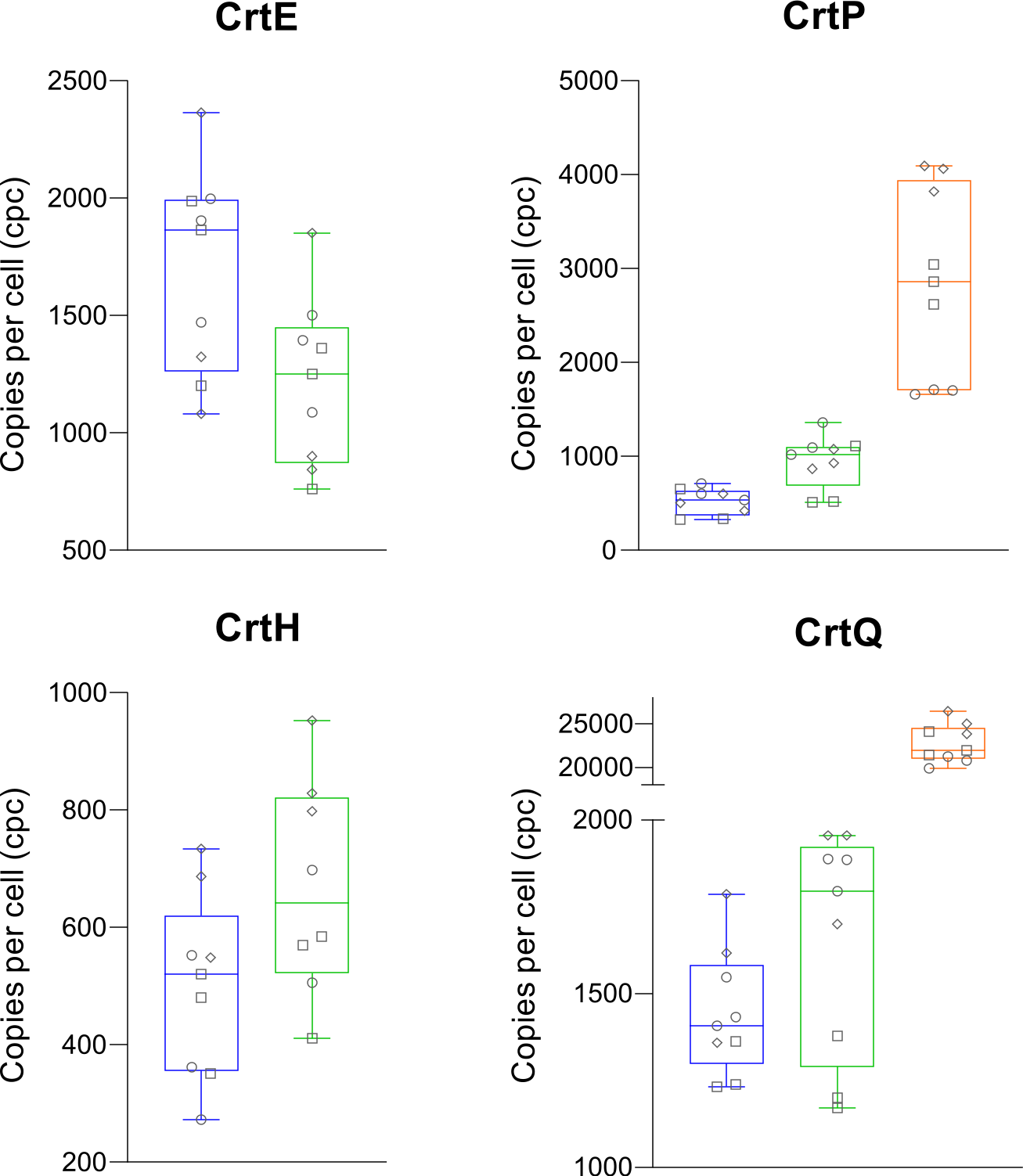 | 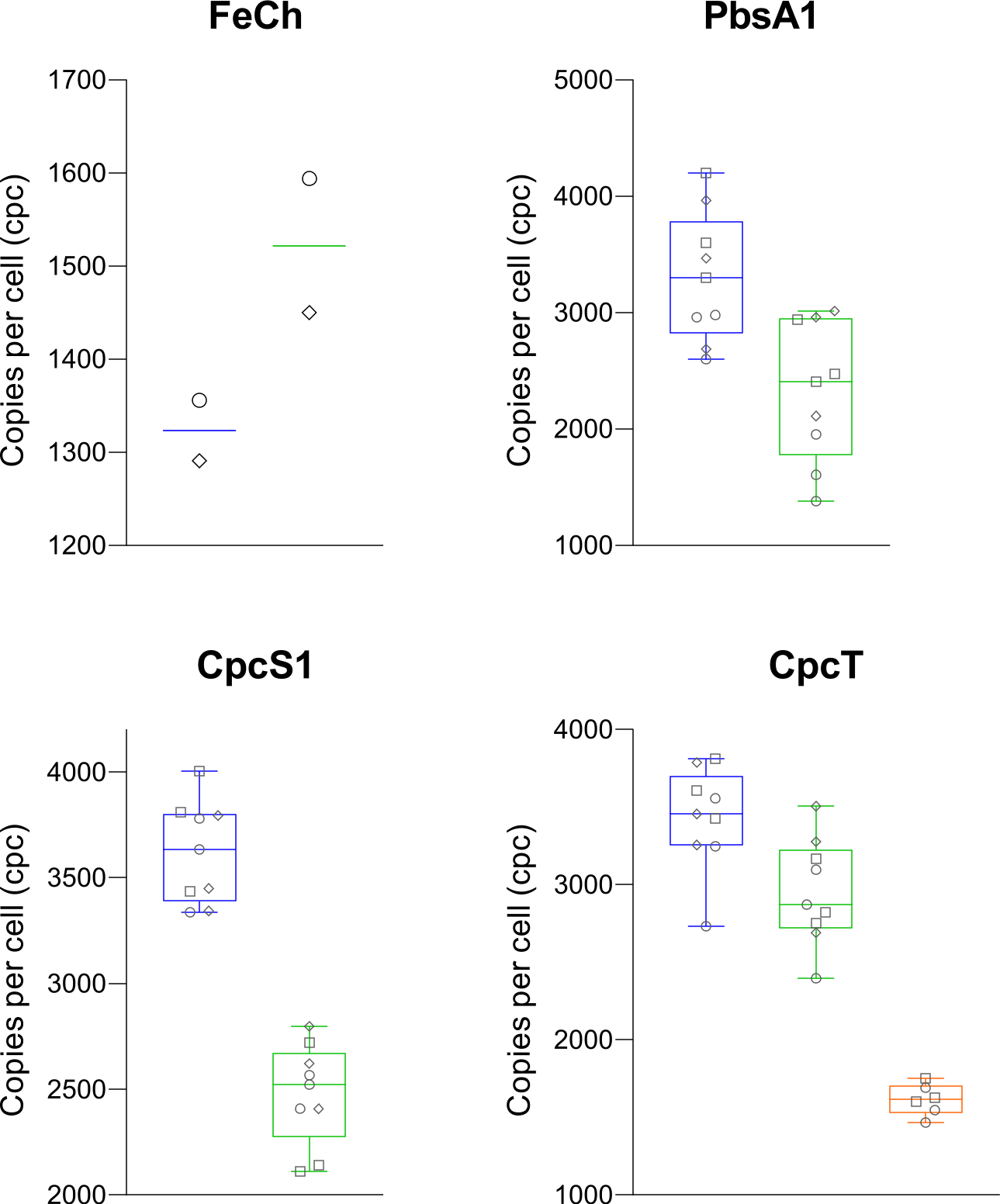 |

**Figure S4** Boxplots showing copies per cell (cpc) distributions of enzymes and auxiliary proteins involved in biosynthesis. Data-points for biological replicates 1, 2 and 3 are represented by circles, squares and diamonds respectively, with 3 technical repeats of each. The boxplots, showing interquartile range, median and minimum/maximum, are colored according to the quantification methods used: SIL-DDA (magenta, with quantotypic peptides identified), iBAQ-DDA (blue), Top3-DDA (green) and Top3-DIA (orange), using the data-points tabulated in Supplementary Data Sets S3-S6 respectively. Note that Fd is quantified by Top2, not Top3, because its trypsin cleavage sites only generate two proteotypic peptides (see Supplementary Data Sets S5 and S6); Fd abundance may therefore be under-estimated.

| 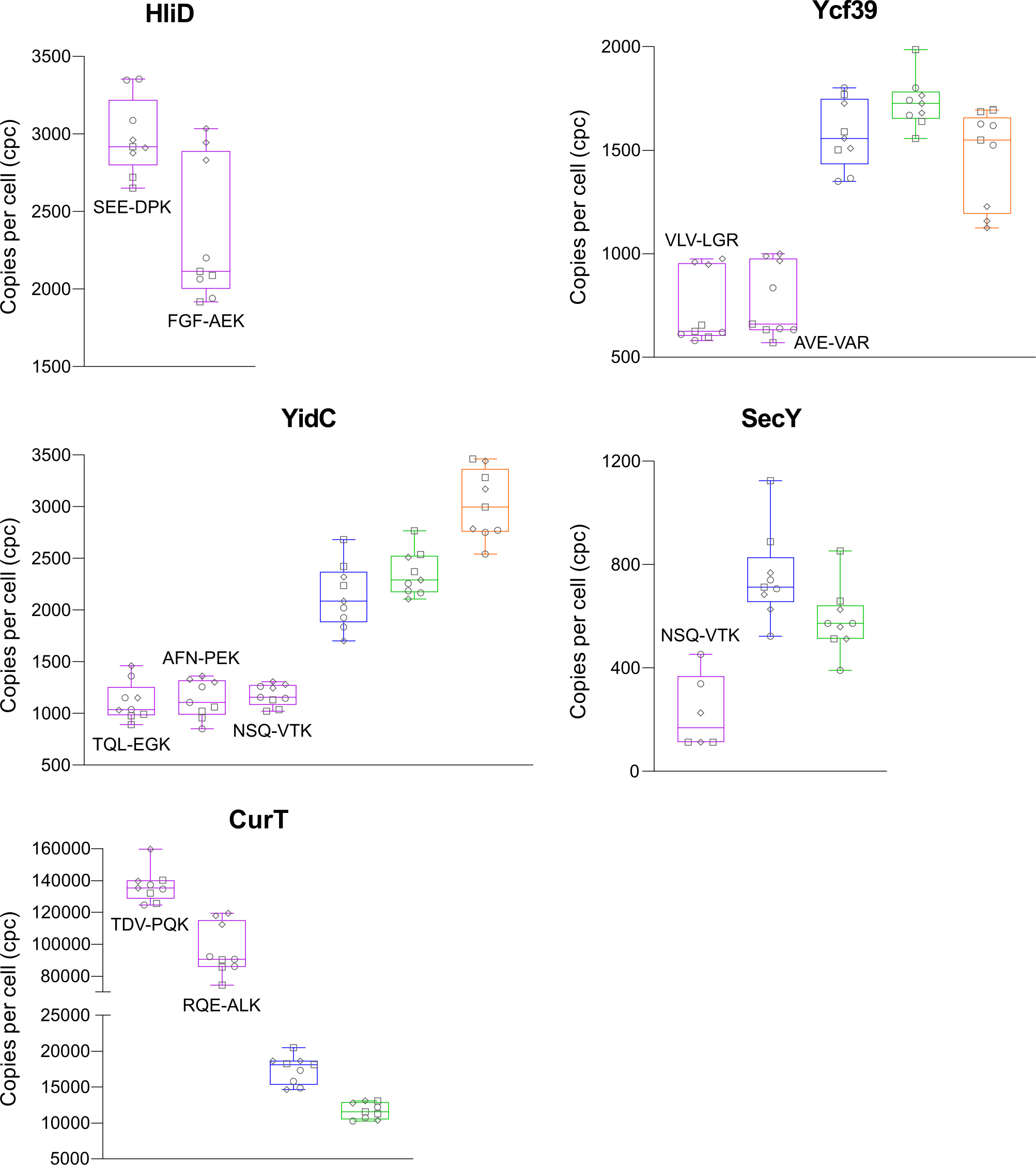 | 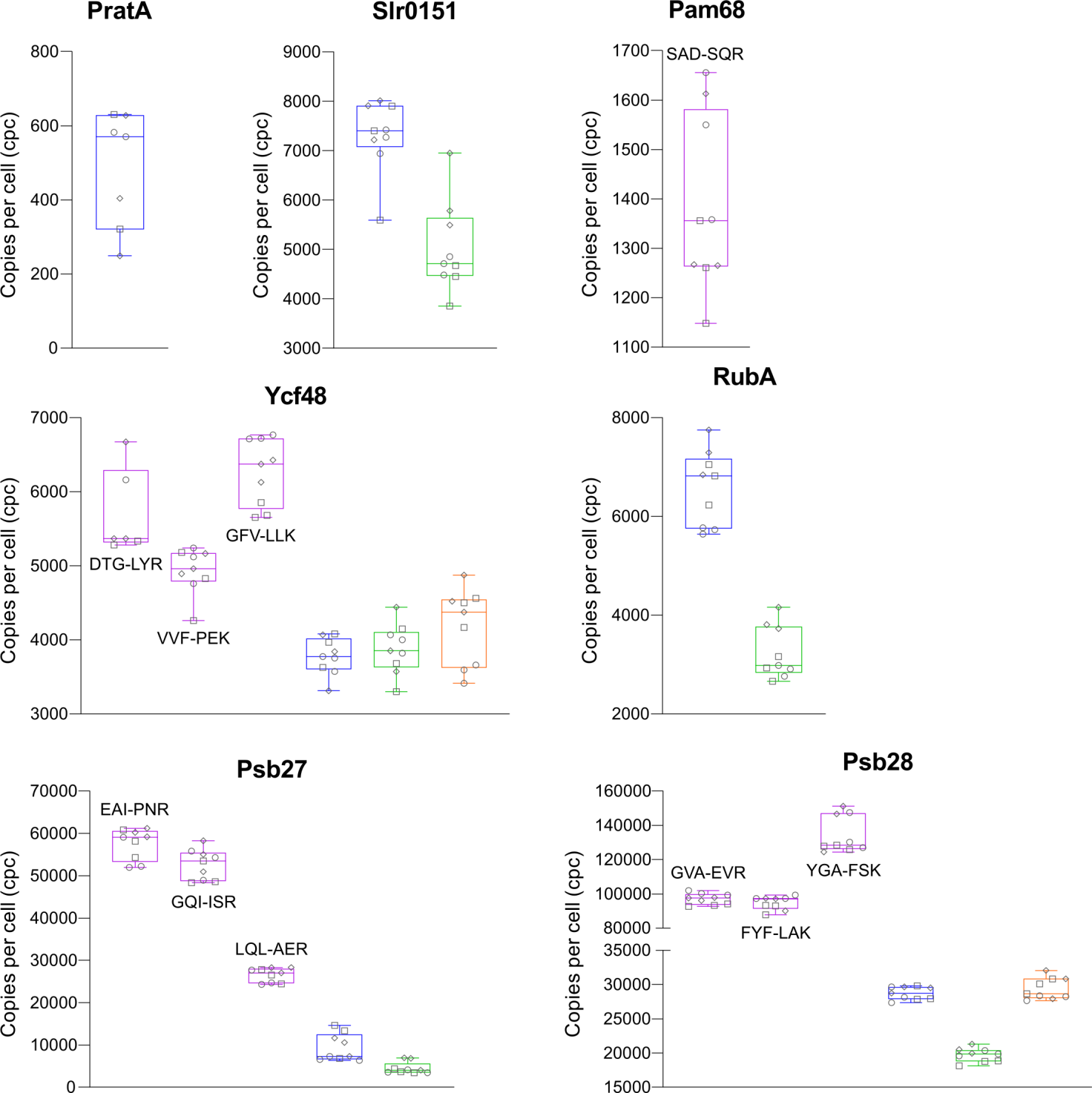 |
| --- | --- |
| 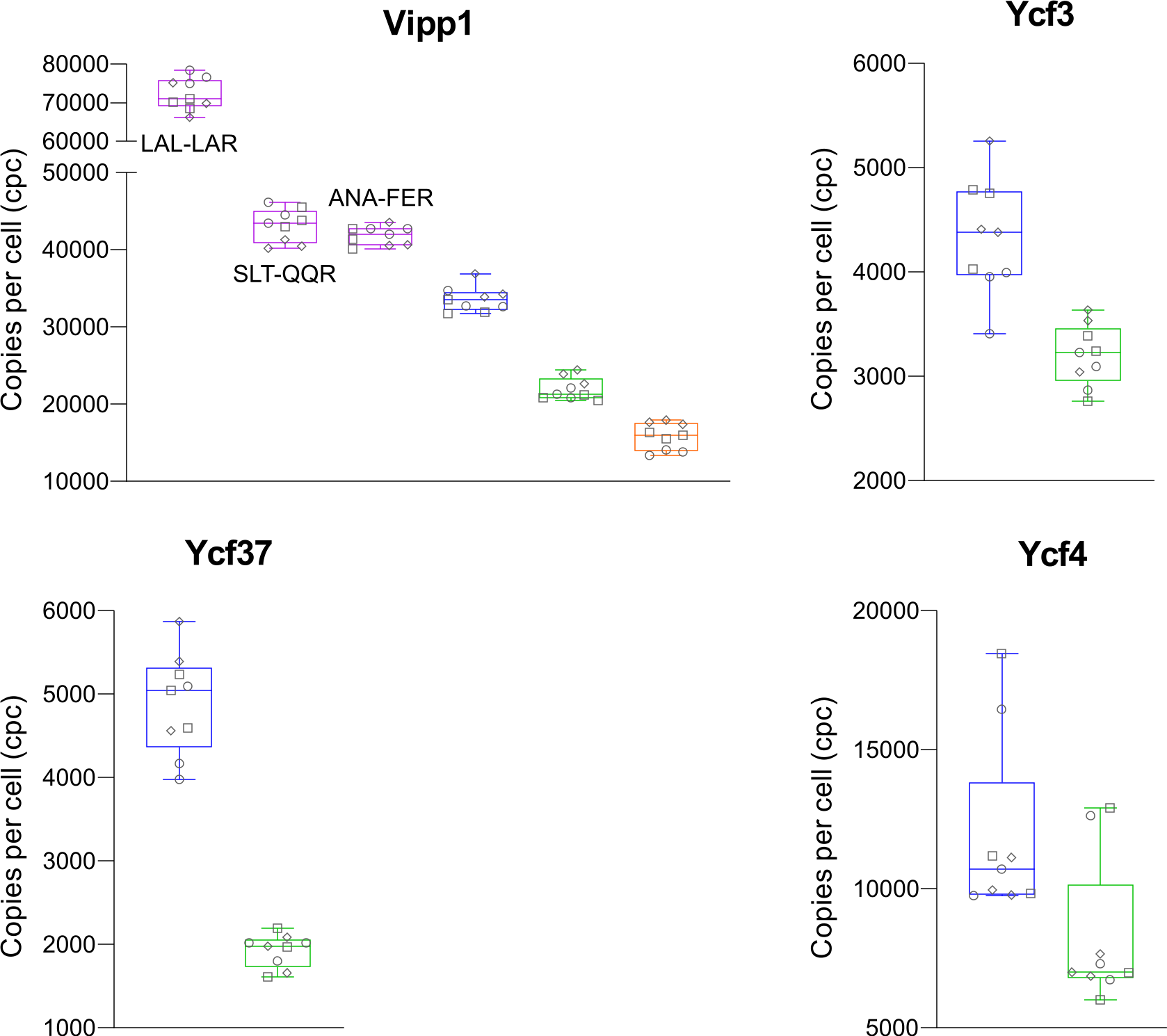 | 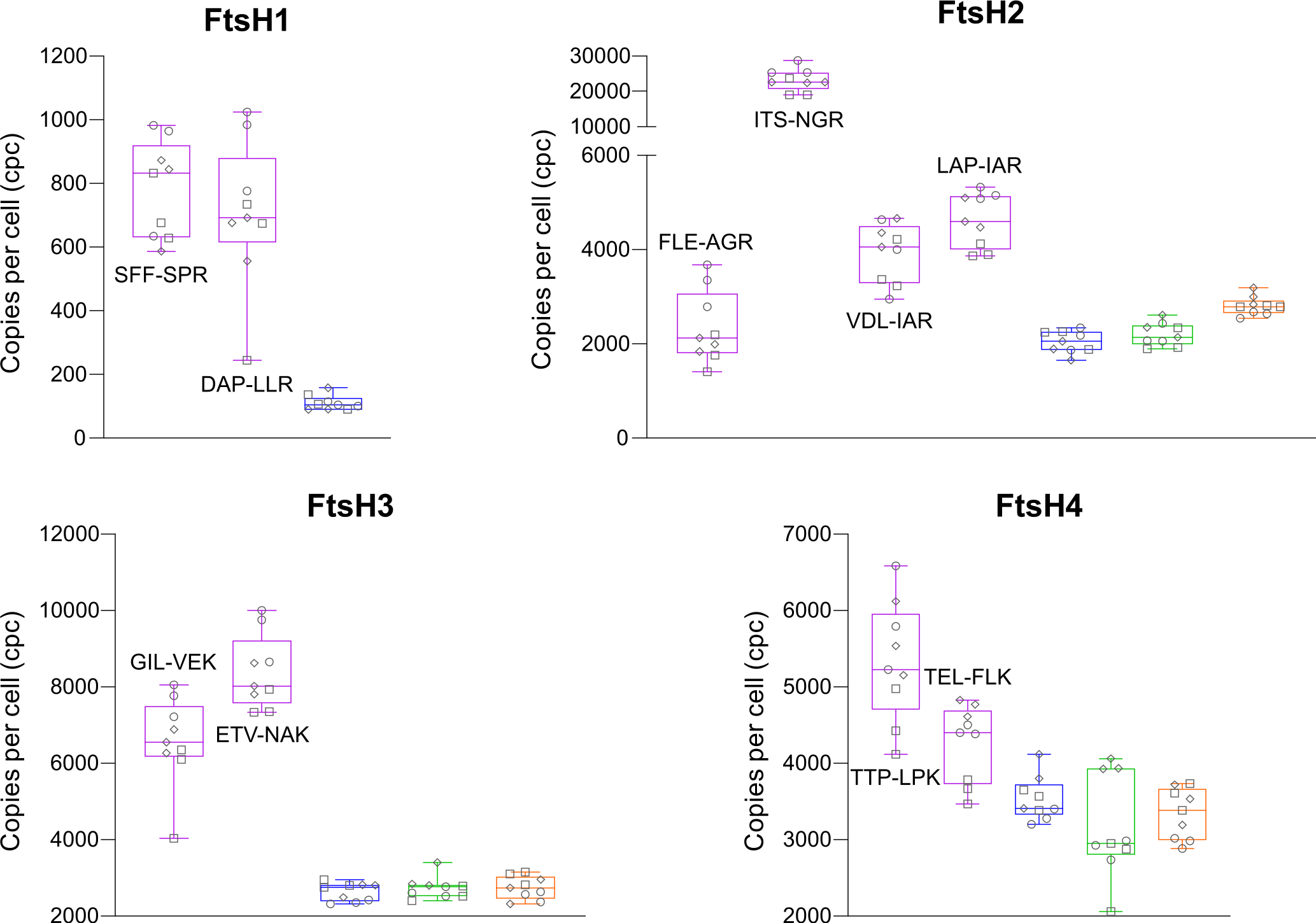 |

**Figure S5** Boxplots showing copies per cell (cpc) distributions of assembly factors and enzymes involved in thylakoid membrane biogenesis and photosystem assembly/repair. Data-points for biological replicates 1, 2 and 3 are represented by circles, squares and diamonds respectively, with 3 technical repeats of each. The boxplots, showing interquartile range, median and minimum/maximum, are colored according to the quantification methods used: SIL-DDA (magenta, with quantotypic peptides identified), iBAQ-DDA (blue), Top3-DDA (green) and Top3-DIA (orange), using the data-points tabulated in Supplementary Data Sets S3-S6 respectively.
